# Dopamine facilitates the response to glutamatergic inputs in astrocyte cell models

**DOI:** 10.1371/journal.pcbi.1012688

**Published:** 2024-12-16

**Authors:** Thiago Ohno Bezerra, Antonio C. Roque

**Affiliations:** Department of Physics, School of Philosophy, Sciences and Letters of Ribeirão Preto, University of São Paulo, Ribeirão Preto, São Paulo, Brazil; Instytut Biologii Doswiadczalnej im M Nenckiego Polskiej Akademii Nauk, POLAND

## Abstract

Astrocytes respond to neurotransmitters by increasing their intracellular Ca^2+^ concentration (Ca^2+^ signals). While glutamate released by neurons trigger Ca^2+^ signals through IP_3_- and glutamate transporter-dependent mechanisms, dopamine released in distant sites activates astrocytes via dopaminergic receptors. However, little is known about the modulatory effects of dopamine on glutamate-evoked astrocytic activity. To investigate this question, we developed multi-compartment, conductance-based astrocyte models with three distinct morphologies: unipolar; bipolar; and bifurcated-terminal. Glutamate induced localized responses, while dopamine activated all compartments. In the unipolar model, global dopaminergic stimulation reduced the threshold frequency of glutamatergic stimulation required to activate Ca^2+^ signals. Phase-plane analysis of a simplified version of this model revealed that Ca^2+^ signals are influenced by compartment radius and neurotransmitter type. Morphology significantly influenced glutamate-dopamine interactions. In the bipolar model, glutamatergic stimulation in one process minimally affected the other. Conversely, in the bifurcated-terminal model, where a single process bifurcates into two secondary processes, high-frequency glutamatergic stimulation in one secondary process evoked Ca^2+^ signals in the other. Dopamine further facilitated this latter cross-process interaction by lowering the glutamatergic stimulation frequency needed to elicit Ca^2+^ signals in the adjacent secondary process. These findings suggest that dopamine enhances the initiation and propagation of glutamate-evoked Ca^2+^ signals, with the extent of propagation depending on astrocytic morphology and the spatial distribution of glutamatergic inputs.

## Author summary

Astrocytes can promote synchronization of neural populations and are involved in information processing and mediation of cognitive functions. In a tripartite synapse, neurotransmitters released by the presynaptic neuron activate receptors on the astrocyte membrane and increase intracellular Ca^2+^ concentration (called Ca^2+^ signals). Astrocytes can show both localized responses triggered by local synaptic (e.g. glutamatergic) input and global responses elicited by neuromodulators like dopamine. To investigate how dopamine influences glutamatergic-evoked astrocyte activity and how these neurotransmitters could be associated to local and global astrocytic responses, we developed compartmental astrocyte models with different geometries that capture generic morphological properties, like processes that emanate from the soma and processes that bifurcate into two secondary processes. Both dopamine and glutamate trigger Ca^2+^ signals in our astrocyte models. By comparing the frequency and location of the Ca^2+^ signals triggered in the different models, our simulation results reveal that dopamine generally enhances the response to local glutamatergic input and facilitates the communication between astrocytic secondary processes.

## Introduction

Traditionally, astrocytes were believed to fulfill basic neuronal support roles like potassium regulation, metabolic maintenance, and neurotransmitter recycling [1]. However, recent research has unveiled a far more dynamic role for these cells. Beyond mere support, astrocytes now appear as active regulators of neural activity, participating in information processing, encoding, and even influencing cognitive functions and behavior. Studies demonstrate their involvement in diverse functions, from controlling motor behavior in zebrafish [2] to responding to arousal and noradrenaline release [3], and modulating goal-directed behavior in the prefrontal cortex of mice [4]. Furthermore, astrocytes play an important role in mediating the psychomotor effects of amphetamine in mice [5] and the impact of cannabidiol on long-term memory [6]. Neurons and astrocytes have a bidirectional communication, forming the so called tripartite synapse, in which a fine astrocytic process ensheathes a synaptic terminal [7]. Astrocytes release neuroactive molecules called gliotransmitters, such as glutamate, ATP and D-serine [1, 5, 8], which modulate synaptic transmission, increase neuron excitability, influence synaptic plasticity, and promote neuronal synchronization [9–14].

Neurons affect astrocytes by elevating the intracellular Ca^2+^ concentration, a phenomenon termed as ‘Ca^2+^ signal’ or ‘Ca^2+^ transient’. When neurotransmitters are released by neurons, they activate G protein-coupled receptors in astrocytes, leading to an increase in intracellular Ca^2+^ levels. Glutamate activates the metabotropic glutamate receptor (mGluR), and noradrenaline and dopamine activate the D_1_ and α_1_ receptors. The activation of these receptors promote the synthesis of IP_3_ by phospholipase C (PLC) [3, 5, 9, 15–18]. IP_3_ then activates IP_3_ receptors on the endoplasmic reticulum (ER) membrane, causing the release of Ca^2+^ from ER internal stores and increasing cytosolic Ca^2+^ concentration. To regulate the intracellular Ca^2+^ concentration, Ca^2+^ is subsequently pumped back into the ER by the sarco/endoplasmic reticulum Ca^2+^-ATPase (SERCA) [1]. Additionally, Ca^2+^ leakage through channels in the ER membrane further modulates astrocytic Ca^2+^ levels. A second mechanism by which glutamate influences astrocytic Ca^2+^ concentration involves the activation of the glutamate transporter (GluT) together with the Na^+^/Ca^2+^-exchanger (NCX) [17, 19]. In this process, Na^+^ is transported into the astrocyte during each GluT cycle. To maintain Na^+^ equilibrium, NCX promotes the efflux of Na^+^ and the influx of Ca^2+^.

As shown by Bindocci *et al*. [20], astrocytes exhibit two distinct patterns of activity: localized activity and a global increase in intracellular Ca^2+^, often associated with the simultaneous activation of multiple astrocytic branches. Challenging previous beliefs, Bindocci and colleagues showed that astrocytic activity primarily occurs locally, with Ca^2+^ signals originating in distant regions seldom reaching the cell body. Global events, on the other hand, likely stem from stimuli that engage extensive astrocytic regions. Yet, the mechanisms triggering these global events, their functions, and their interplay with local responses remain elusive.

The local and global responses may be linked to different mechanisms of neurotransmitter release. For instance, glutamate released by presynaptic cells in tripartite synapses serves as a primary source of glutamatergic input to astrocytes, likely driving localized astrocytic responses [7, 21]. In contrast, neuromodulators like dopamine and noradrenaline, which diffuse widely in the extracellular space, are capable of activating broader astrocytic regions and modulating overall astrocytic activity [22, 23]. Dopamine has an important role in regulating brain functions such as control of motor behavior, signaling stimulus salience, working memory, and motivation [24–27]. Some psychiatric disorders associated with alterations in dopaminergic transmission, for example schizophrenia and attention-deficit/hyperactivity disorder, cause deficits in executive functions, including working memory [28]. Since astrocytes are influenced by dopaminergic signaling [5, 18, 29], have been implicated in these cognitive functions [30, 31], and are affected by psychiatric conditions [32], it is plausible that dopamine could exert its influence on these processes partly via astrocytic mechanisms.

Although crucial for understanding astrocytic function within neural systems, addressing these questions poses challenges in monitoring entire astrocytes and isolating stimulation to specific regions. Hence, leveraging computational and mathematical modeling techniques becomes important to help elucidating these questions. Li and Rinzel [33] pioneered a model describing the relationship between intracellular Ca^2+^ and IP_3_. Building upon this framework, De Pittà and colleagues [16] developed a model wherein mGluR activation of the IP_3_ pathway induces Ca^2+^ release from the ER. Oschmann and colleagues [19] expanded this research line by incorporating the GluT-dependent mechanism into their astrocytic model, elucidating how the two glutamatergic mechanisms are linked to the ER-cytosol volume ratio. They showed that in fine astrocytic processes, the GluT mechanism exerts a stronger influence on Ca^2+^ dynamics, while in thicker branches, the mGluR mechanism dominates. Other investigations have employed compartmental models [34] or detailed data-driven spatial templates [39] to study astrocytic function. In particular, Gordleeva *et al*. [34] showed that the transient enhancement of synaptic transmission by Ca^2+^-dependent glutamate release from astrocytes could promote neural synchrony. Similarly, Verisokin *et al*. [40] developed a computational model in which astrocytes respond to synaptic and noradrenergic inputs, showing that noradrenaline can promote broad responses in astrocytes. These models are remarkable insofar as they put emphasis on the potential critical role of spatiotemporal dynamics of astrocytic calcium signaling in shaping network states as otherwise supported by spiking neuron-astrocyte network models [35–37] and experiments (reviewed in Oliveira and Araque [38], and Santello *et al*. [30]).

However, to the best of our knowledge, no model has yet investigated how dopaminergic input can modulate astrocytic response to glutamatergic stimulation. In this work, we employed a compartmental model of an astrocyte with a soma and a single process to investigate how synaptic glutamatergic (local) and modulatory dopaminergic (global) inputs interact and influence the intracellular Ca^2+^ concentration. In agreement with experimental findings [20], our simulation results show the emergence of a compartmentalized dynamics in which Ca^2+^ signals remain mostly confined to the mid-process region. Furthermore, our results show that dopamine facilitates the response to glutamatergic inputs arriving at distal regions, and that high-frequency dopaminergic stimulation can produce Ca^2+^ signals throughout the entire extent of the astrocytic process. Harnessing the time scale difference between model variables, we developed a two-variable version of this model which allows a mathematical study of the effects of compartment thickness and neurotransmitter type on the intracellular Ca^2+^ and IP_3_ dynamics, offering insights into the results of the full compartmental model. Finally, we created model variations with branched morphologies, with two processes originating from the soma or a process that bifurcates into two secondary processes at its end point, to explore how astrocytic branch geometry influences compartmentalized dynamics. Our results suggest that morphology affects glutamate-induced Ca^2+^ activity more than dopamine-induced activity.

## Methods

We developed a compartmental astrocyte model based on previous computational models available in the literature [16, 19, 34, 42]. The compartmental model is composed of a spherical soma with interconnected unit-length cylindrical compartments representing a linear astrocytic process. The following description of the compartmental model is divided into three subsections: first, we outline the equations for a single compartment, without accounting for coupling with others. Next, we introduce a simplified, two-variable version of the model. Lastly, we explain the coupling of compartments and present the basic model and its branched variations that we used to study the effect of process geometry.

### Detailed compartment model

**Fig 1:**
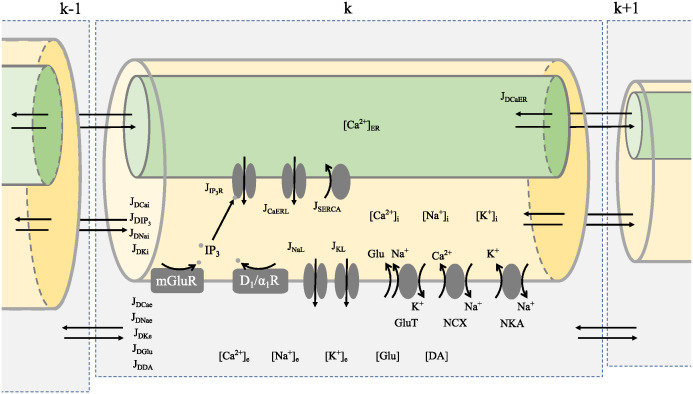
Generic astrocyte compartment showing the model variables. Each compartment is subdivided into intracellular space (yellow cylinder), ER (green cylinder) and extracellular space (gray box). The intracellular Ca^2+^ concentration is influenced by activation of IP_3_ receptors in the ER membrane, Ca^2+^ leakage from ER, SERCA pump uptake into ER, current through NCX and Ca^2+^ diffusion between intracellular compartments (*J*_*D*Cai_). The Ca^2+^ concentration in ER is governed by activation of IP_3_ receptors, leakage from ER, SERCA pump into ER and Ca^2+^ diffusion between ER compartments (*J*_*D*CaER_). The extracellular Ca^2+^ concentration is affected by the current through NCX and Ca^2+^ diffusion between extracellular compartments (*J*_*D*Cae_). The IP_3_ concentration is determined by the activation of mGluR and D_1_/α_1_ receptors and the IP_3_ diffusion between intracellular compartments ($J_{D{\text{IP}}_{3}}$). Extracellular glutamate ([Glu]) also promotes influx of Na^+^ and efflux of K^+^ through a GluT-dependent mechanism. The intracellular and extracellular Na^+^ concentrations are determined by the leak current density through Na^+^ channels (*J*_NaL_), the pump current through NKA and by Na^+^ diffusion (intracellular: *J*_*D*Nai_; extracellular: *J*_*D*Nae_). Similarly, the intracellular and extracellular K^+^ concentrations are controlled by the K^+^ leak current density (*J*_KL_), the NKA current and K^+^ diffusion (intracellular: *J*_*D*Ki_; extracellular: *J*_*D*Ke_). The extracellular concentrations of glutamate ([Glu]) and dopamine ([DA]) are affected by glutamate and dopamine diffusion in the extracellular space (*J*_*D*Glu_ and *J*_*D*DA_, respectively).

The generic structure of a compartment is shown in Fig 1. The compartment is subdivided into three subspaces connected by current densities between them: a) intracellular space; b) extracellular space; and c) ER. We assumed that the intra- and extracellular spaces of the compartment have equal volumes. Since the volume of the ER varies along the astrocytic processes, to adjust the current that flows through the ER to the intracellular space, we implemented a factor *r*_ER_ representing the ratio between the volumes of ER and cytosol [19, 34]. This ratio is dependent on the surface-to-volume ratio of the compartment, *A*/*V*, where *A* and *V* are the area and volume of the compartment, respectively [43]. The factor *r*_ER_ is calculated as:

$$\begin{array}{c} r_{ER}=ae^{-{\left(bA/V\right)}^{c}}, \end{array}$$ (1)

where the parameters *a* = 0.15, *b* = 0.073 μm and *c* = 2.34 were fitted using experimental data [43].

The following variables were considered in the astrocytic compartment: intracellular and extracellular Ca^2+^ concentrations ([Ca^2+^]_*i*_ and [Ca^2+^]_*e*_, respectively); ER Ca^2+^ concentration ([Ca^2+^]_ER_); intracellular IP_3_ concentration ([IP_3_]); the fraction of open IP_3_ receptors (*h*); intracellular and extracellular Na^+^ concentrations ([Na^+^]_*i*_ and [Na^+^]_*e*_, respectively); intracellular and extracellular K^+^ concentrations ([K^+^]_*i*_ and [K^+^]_*e*_, respectively); the compartment potential (*V*); and extracellular glutamate ([Glu]) and dopamine ([DA]) concentrations.

There are two distinct spatial glutamatergic mechanisms influencing the intracellular Ca^2+^ concentration in our model [19]: the mGluR pathway, which is an IP_3_-dependent mechanism, and the GluT pathway (see Fig 1). In proximal compartments, since the value of *r*_ER_ is higher, the IP_3_-dependent mechanism has a stronger effect on the intracellular Ca^2+^. On the other hand, in distal compartments, which have lower *r*_ER_ values, the GluT mechanism has a stronger effect. Since dopamine activates both the dopaminergic D_1_ and the noradrenergic α_1_ receptors on the astrocyte membrane in the prefrontal cortex [18, 29], we describe the receptors activated by dopamine just as dopaminergic receptors.

The intracellular [Ca^2+^]_*i*_, extracellular [Ca^2+^]_*e*_ and intra-ER [Ca^2+^]_ER_ Ca^2+^ concentrations obey the following equations [16, 44]:

$$\frac{d{\left[{\text{Ca}}^{2+}\right]}_{i}}{dt}=\frac{A}{VF}J_{\text{NCX}}+\frac{A\sqrt{r_{\text{ER}}}}{VF}\left(J_{{\text{IP}}_{3}\text{R}}-J_{\text{SERCA}}+J_{\text{CaERL}}\right),$$ (2)

$$\frac{d{\left[{\text{Ca}}^{2+}\right]}_{e}}{dt}=-\frac{A}{VF}J_{\text{NCX}},$$ (3)

$$\frac{d{\left[{\text{Ca}}^{2+}\right]}_{\text{ER}}}{dt}=\frac{A\sqrt{r_{\text{ER}}}}{VFr_{\text{ER}}}\left(-J_{{\text{IP}}_{3}\text{R}}+J_{\text{SERCA}}-J_{\text{CaERL}}\right),$$ (4)

where *J*_NCX_ is the NCX current density, $J_{{\text{IP}}_{3}\text{R}}$ is the current density through the IP_3_R channel on the ER, *J*_SERCA_ is the current density through SERCA, *J*_CaERL_ is the leak current density from ER. The expressions for current densities $J_{{\text{IP}}_{3}\text{R}}$, *J*_SERCA_, *J*_CaERL_, and *J*_NCX_, are given in the S1 Text (equations (S1)–(S3), and (S6), respectively). The factors $A\sqrt{r_{\text{ER}}}$ and *Vr*_ER_ represent the area and the volume of ER.

The intracellular and extracellular Na^+^ and K^+^ concentrations are modeled as:

$$\frac{d{\left[{\text{Na}}^{+}\right]}_{i}}{dt}=\frac{A}{VF}\left(3J_{\text{GluT}}-3J_{\text{NKA}}-3J_{\text{NCX}}-J_{\text{NaL},j}\right)$$ (5)

$$\frac{d{\left[{\text{Na}}^{+}\right]}_{e}}{dt}=-\frac{A}{VF}\left(-3J_{\text{GluT}}+3J_{\text{NKA}}+3J_{\text{NCX}}+J_{\text{NaL}}\right),$$ (6)

$$\frac{d{\left[{\text{K}}^{+}\right]}_{i}}{dt}=\frac{A}{VF}\left(-J_{\text{GluT}}+2J_{\text{NKA}}-J_{\text{KL}}\right)$$ (7)

$$\frac{d{\left[{\text{K}}^{+}\right]}_{e}}{dt}=\frac{A}{VF}\left(J_{\text{GluT}}-2J_{\text{NKA}}+J_{\text{KL}}\right),$$ (8)

where *J*_GluT_ is the current density through the glutamate transporters (equation (S4)), *J*_NKA_ is the current density through NKA pumps (equation (S5)), *J*_NaL_ and *J*_KL_ are the leak current density through the Na^+^ and K^+^ channels (equations (S7)–(S8)). The integer factors multiplying the current densities *J*_GluT_, *J*_NKA_ and *J*_NCX_ represent the number of ions carried by these currents in each cycle [19].

The dependence of the compartment potential *v* on the ionic current densities is described by the following equation:

$$\begin{array}{cc} \frac{dv}{dt} & =-\frac{1}{C_{m}}(-2J_{{\text{IP}}_{3}\text{R}}+2J_{\text{SERCA}}-2J_{\text{CaERL}}+J_{\text{NCX}}-\\ & 2J_{\text{GluT}}+J_{\text{NKA}}+J_{\text{NaL}}+J_{\text{KL}}), \end{array}$$ (9)

where the integer factors multiplying each current density represent the net current or the number of ions carried per cycle [19].

The intracellular IP_3_ concentration is governed by the equation [16, 44]:

$$\begin{array}{cc} \frac{d[{\text{IP}}_{3}]}{dt} & =S_{\text{PLC}\beta_{\text{Glu}}}+S_{\text{PLC}\beta_{\text{DA}}}+S_{\text{PLC}\delta }-D_{{\text{IP}}_{3}-3\text{K}}-D_{\text{IP}-5\text{P}}, \end{array}$$ (10)

where $S_{\text{PLC}\beta_{\text{Glu}}}$ represents the synthesis of IP_3_ by the activation of mGluR (equation (S9)), $S_{\text{PLC}\beta_{\text{DA}}}$ is the synthesis of IP_3_ by the activation of D_1_/α_1_ receptors (equation (S10)), *S*_PLC*δ*_ is the synthesis of IP_3_ by PLC*δ* (equation (S11)), $D_{{\text{IP}}_{3}-3\text{K}}$ is the degradation of IP_3_ by the IP_3_-3K (equation (S12)), *D*_IP–5P_ is the degradation of IP_3_ by IP-5P (equation (S13)).

The fraction of open IP_3_R channels in compartment *j* is given by:

$$\frac{dh}{dt}=a_{2}(d_{2}\frac{\left[{\text{IP}}_{3}\right]+d_{1}}{\left[{\text{IP}}_{3}\right]+d_{3}})\left(1-h\right)-{\left[{\text{Ca}}^{2+}\right]}_{i}h,$$ (11)

where *a*_2_ = 0.2 μM^-1^ s^-1^ is the Ca^2+^ inhibition constant, *d*_2_ = 1.049 μM is the Ca^2+^ dissociation constant, *d*_3_ = 0.9434 μM is the receptor dissociation constant of IP_3_ and *d*_1_ = 0.13 μM is the IP_3_ dissociation constant.

The extracellular concentrations of glutamate and dopamine are modeled as:

$$\begin{array}{c} \frac{d[\text{Glu}]}{dt}=-G_{\text{Glu}}\left[\text{Glu}\right]+\rho_{\text{Glu}}\delta \left(t-t_{\text{spike}}\right), \end{array}$$ (12)

$$\begin{array}{c} \frac{d[\text{DA}]}{dt}=-G_{\text{DA}}\left[\text{DA}\right]+\rho_{\text{DA}}\delta \left(t-t_{\text{spike}}\right), \end{array}$$ (13)

where *t*_spike_ is the presynaptic spike time and represents glutamate or dopamine release events, *G*_Glu_ = 100 s^-1^ and *G*_DA_ = 4.201 s^-1^ are, respectively, the inverses of the time constants of glutamate and dopamine decay, and *ρ*_Glu_ = 0.5 μM and *ρ*_DA_ = 3 μM are, respectively, the amounts of glutamate and dopamine released in each presynaptic neuron spike. These parameters were adjusted to reproduce experimental measurements of extracellular concentrations of glutamate and dopamine, as well as the responses of astrocytes to these neurotransmitters [16, 22, 34, 45–47]. For convenience, all model parameters along with the references from which they were obtained are provided in Table A of the S1 Table. Further details on the parameter adjustments made are also given in the S1 Table.

### Simplified compartment model

Astrocytes respond to changes in Ca^2+^ ion concentration on a scale of seconds, whereas fluctuations in the levels of Na^+^ and K^+^ ions, membrane potential *V*, the proportion of IP_3_ open channels, and extracellular Ca^2+^ occur in milliseconds. To distill the complex biophysical model down to its core mechanisms, we developed a simplified model of the astrocytic compartment focusing on Ca^2+^ and IP_3_ dynamics. The simplified model derivation and its detailed description are provided in the section ‘Simplified Model’ in the S1 Text.

In short, we replaced the rapidly changing variables ([Na^+^]_*i*_, [Na^+^]_*e*_, [K^+^]_*i*_, [K^+^]_*e*_, *v* and [Ca^2+^]_*e*_) by their respective resting values. We also approximated [Ca^2+^]_ER_ as a function of [Ca^2+^]_*i*_. Additionally, because *h* does not fluctuate significantly, we replaced it by its average value. These substitutions allowed us to simplify the detailed compartment model into a model with two dimensionless variables, namely *c* and *i*, which represent the intracellular concentrations of Ca^2+^ and IP_3_, respectively (see the S1 Text for details about the rescaling and the simplified model). After this simplification, the dynamics of *c* and *i* are described by:

$$\frac{\text{d}c}{\text{d}t}=\frac{A}{FV}J_{\text{NCX}}+\frac{A\sqrt{{\text{r}}_{\text{ER}}}}{FV}(J_{{\text{IP}}_{3}\text{R}}-J_{\text{SERCA}}+J_{\text{CaERL}}),$$ (14)

$$\frac{\text{d}i}{\text{d}t}=S_{\text{PLC}\beta_{\text{Glu}}}+S_{\text{PLC}\beta_{\text{DA}}}+S_{\text{PLC}\delta }-D_{{\text{IP}}_{3}-3\text{K}}-D_{\text{IP}-5\text{P}},$$ (15)

where, with the exception of *J*_NCX_, the current densities are the same as for the detailed astrocyte model. The expressions for the Ca^2+^ currents and IP_3_ synthesis and degradation in the simplified model are given in S1 Text. The extracellular concentrations of glutamate and dopamine in the simplified model were also rescaled and treated as dimensionless parameters *g* and *d*, respectively.

The simplified model is a two-dimensional system that offers certain advantages over the detailed model. It can be simulated computationally at a lower cost, and its behavior can be analyzed analytically in the (*c*-*i*) phase plane using tools from dynamical systems theory.

### Compartmental coupling and cell morphologies

As shown in Fig 1, the intracellular, extracellular, and ER subspaces of consecutive compartments are coupled by diffusive fluxes of Ca^2+^, Na^+^, K^+^, IP_3_, glutamate and dopamine that change the concentrations of these ions/molecules within them. Therefore, in the above equations for the rates of change of these ions/molecules extra terms must be added to account for the full compartmental model. The generic expression for the rate of change of the concentration of ion/molecule X in compartment *j* due to diffusion of X from a neighboring compartment *k* is given by [48]:

$$\begin{array}{c} (\frac{d{\left[\text{X}\right]}_{j}}{dt}{)}_{\text{diff}}=F_{\text{X}}({\left[\text{X}\right]}_{k}-{\left[\text{X}\right]}_{j}), \end{array}$$ (16)

where *F*_X_ (in s^-1^) is the coupling strength between compartments *j* and *k* for X. The coupling strength depends on the geometry of the compartments and the diffusion coefficient *D*_X_. The derivation of its expression and the values for the distinct types of compartmental coupling are given in the text that accompanies the parameter values tables in the S1 Table.

**Fig 2:**
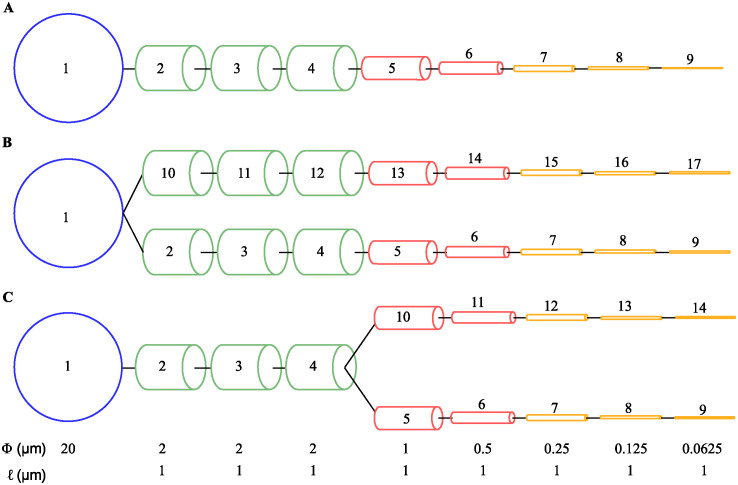
Astrocyte morphological models. (**A**) Unipolar morphology. (**B**) Bipolar morphology. (**C**) Bifurcated-terminal morphology. The compartments are numbered in crescent order from soma to distal compartments. The dimensions of the soma (blue), proximal (green), intermediate (red) and distal (yellow) compartments are the same for all three morphologies, and are indicated at the bottom as Φ (radius) and *ℓ* (length).

We used the compartment model and the coupling scheme above to construct compartmental astrocyte models with three different morphologies:

- Unipolar morphology: a soma with a single process emanating from it, without branching (Fig 2A);
- Bipolar morphology: a soma with two equal processes emanating from it, without further branching (Fig 2B);
- Bifurcated-terminal morphology: a soma with a single process that bifurcates into two equal secondary processes at its end (Fig 2C);

The somatic compartment is a sphere with radius of 20 μm, and the remaining compartments are cylinders with unitary length *ℓ* = 1 μm and radii varying from 2 μm, for compartments coupled to soma, to 0.0625 μm, for the most distal compartments (Fig 2). These morphologies were selected to reflect motifs observed in astrocytes, and compartment radii to match the thickness of astrocytic processes observed experimentally [49–52]. The two branched geometries differ in their hierarchical organization. In the bipolar morphology, the two processes emanate directly from the soma and are hierarchically equivalent (they are primary processes). In the bifurcated-terminal morphology, the two secondary processes do not emerge directly from the soma but from a primary process. As a result, they are hierarchically of second order compared to the processes in the bipolar morphology.

### Stimulation protocols

The unipolar model was used as the basic setup for our studies on compartmentalized activity induced in the astrocytic process by stimulation with glutamate alone, dopamine alone, or glutamate and dopamine together. This was done by describing the compartments of the unipolar model using both the full and simplified versions, which allowed us to study the effect of compartment thickness (and its relative position to the soma) on the dynamics of the variables. Subsequently, the models with branched morphologies were used to investigate the effect of astrocytic process geometry on neurotransmitter-induced activity, again considering glutamate alone, dopamine alone, and both together. In particular, since the two branched geometries differ in their hierarchical organization, the study allowed us to identify which configuration enables dopamine to facilitate communication between different primary or secondary processes induced by glutamatergic stimulation of one of the processes.

A summary and a brief description of the experiments done is given below. More details about them are given in the section ‘Stimulation Protocols’ in the S1 Text.

1. Emergence of compartmentalized dynamics: detailed unipolar morphology model.
  (a) Local glutamatergic and global dopaminergic stimulation
  (b) Interaction between local glutamatergic and global dopaminergic stimulation
2. Emergence of compartmentalized dynamics: phase-plane analysis of the simplified model.
  (a) Influence of compartment radius.
  (b) Influence of stimulation type.
3. Interaction between different processes depending on their hierarchical order: detailed nonlinear morphology models.
  (a) Bipolar morphology model.
  (b) Bifurcated-terminal morphology model.

Each stimulation trial had a fixed duration of 100 seconds, with the model starting from its resting state at the beginning of each trial. Glutamatergic and dopaminergic inputs were modeled as independent Poisson spike trains with frequencies *ν*_*g*_ and *ν*_*d*_ applied for the duration of a trial. Glutamatergic stimuli were applied only to the distal compartments of each model (indicated in yellow in Fig 2). This was done to simulate the effect of glutamate released by the presynaptic terminal in a tripartite synapse [7, 21]. Dopaminergic stimulation, on the other hand, was always applied to all compartments of the models, simulating volume transmission [22]. The astrocytic responses were measured by the frequency, location and amplitude of the evoked Ca^2+^ signals.

## Results

We present below the results of the experiments conducted with the astrocyte models described in Methods (see also S1 Text). The results will be presented in the order indicated in the summary of experiments done provided in the previous section.

### Emergence of compartmentalized dynamics

#### Local glutamatergic input

**Fig 3:**
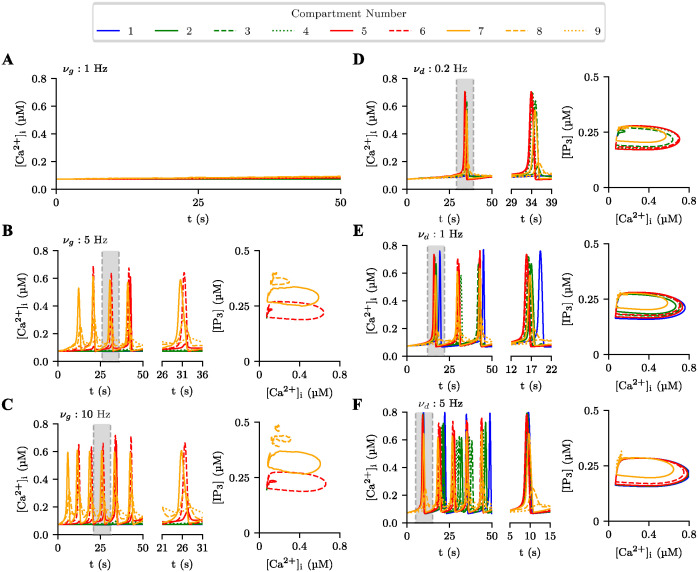
[Ca^2+^]_*i*_ dynamics in the unipolar model with local glutamatergic and global dopaminergic stimulation. Responses in each compartment (color-coded beside the graphs) to distal compartments stimulation by glutamate at frequencies *ν*_*g*_ = 1 Hz (**A**), 5 Hz (**B**), and 10 Hz (**C**), and whole-cell stimulation by dopamine at frequencies *ν*_*d*_ = 0.2 Hz (**D**), 1 Hz (**E**), and 5 Hz (**F**). Details of the Ca^2+^ signals and system oscillations for the shaded regions are shown in the enlarged time scale plots and in the phase plane of IP_3_ vs. Ca^2+^ next to each graph, respectively.

Low-frequency glutamatergic input to the distal compartments (*ν*_*g*_ = 1 Hz) was unable to trigger Ca^2+^ signals in any cellular compartment (Fig 3A). Higher frequency inputs (*ν*_*g*_ = 5 Hz and *ν*_*g*_ = 10 Hz) triggered Ca^2+^ signals in the distal compartments, which propagated to the intermediate region of the astrocytic process (compartments 4, 5, and 6), but not to the proximal regions; no Ca^2+^ signals were detected in compartments 1 (soma) and 2, and only slight fluctuations in [Ca^2+^]_*i*_ were observed in compartment 3 (Fig 3B and 3C). The main change when increasing the stimulation frequency from 5 to 10 Hz was an increase in the frequency of Ca^2+^ signals. Changing the stimulation site to the somatic, proximal, or distal compartments produced similar results, with Ca^2+^ signals triggered only in the stimulated compartments and their neighbors (S1 Fig). These results indicate that there is a minimum frequency required for Ca^2+^ signal generation by glutamatergic stimulation. Although the response to glutamate spread toward other compartments, local glutamatergic input triggers only localized responses.

Ca^2+^ signals generated in the most distal compartments (8 and 9) are low in amplitude and long in duration, while signals generated in the central portion of the process (compartments 4, 5, 6, and 7) are higher in amplitude and shorter in duration. This result indicates that Ca^2+^ signals induced in different compartments have distinct characteristics, being weaker at the tip of the astrocytic process and stronger in the intermediate region of the process.

**Fig 4:**
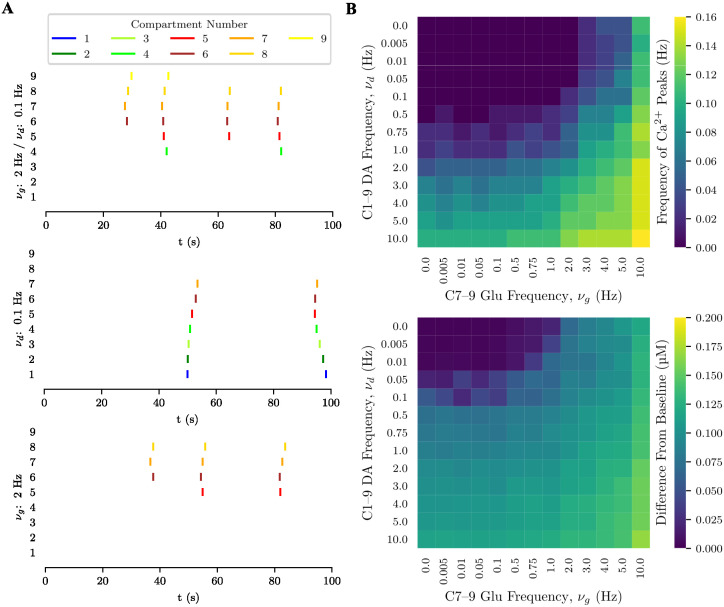
Interaction between dopaminergic and glutamatergic inputs in the unipolar model. (**A**) Raster plot of Ca^2+^ signal activity during a 100 s stimulation trial (time in horizontal axis and compartment number in the vertical axis; vertical bars—color-coded according to process region (yellow: distal; red: intermediate; green: proximal; blue: soma)—indicate Ca^2+^ signals). From top to bottom: joint glutamate and dopamine inputs with *ν*_*g*_ = 2 Hz and *ν*_*d*_ = 0.1 Hz; dopamine input alone with *ν*_*d*_ = 0.1 Hz; and glutamate input alone with *ν*_*g*_ = 2 Hz. (**B**) Frequency (top diagram) and amplitude (bottom diagram) of Ca^2+^ peaks in compartment 9 for different combinations of local glutamatergic and global dopaminergic input frequencies.

A more detailed representation of the compartmentalized sequence of responses to glutamate stimulation can be seen in the raster plot of Fig 4A (bottom plot). The raster plot shows the calcium signals generated in a 100 s stimulation trial with a frequency slightly above the glutamate activation threshold. The first “block” of signals appears around *t* = 40 s, starting from the most proximal distal compartment (7) and then reaching its neighbors (6 and 8). The other two blocks begin from the intermediate compartment 6 and also include compartment 5, but they never reach the most distal compartment (9), the proximal compartments, or the soma.

#### Global dopaminergic input

Unlike glutamatergic stimulation, no minimum input frequency was observed for dopaminergic stimulation to trigger Ca^2+^ signals. Even low-frequency stimulation (*ν*_*d*_ = 0.2 Hz) was able to elicit Ca^2+^ signals (Fig 3D). Interestingly, in this case, the strongest Ca^2+^ signals were generated in the proximal and intermediate compartments (2, 3, 4, 5, and 6), gradually decreasing in amplitude from the beginning of the distal region (compartment 7) to the tip of the process (compartments 8 and 9), where there are only small fluctuations. This suggests that the Ca^2+^ elevations induced by dopamine in the distal compartments were not produced locally but by diffusion from the intermediate compartments.

Dopaminergic inputs at higher frequencies produced calcium signals in all compartments (see Fig 3E for *ν*_*d*_ = 1 Hz and Fig 3F for *ν*_*d*_ = 10 Hz). The somatic response was the highest in amplitude, but the responses in the most distal compartments (8 and 9) remained weak. The frequency of Ca^2+^ signals increased with the rise in dopaminergic stimulation frequency. These results suggest that there is no frequency threshold for the generation of Ca^2+^ signals by dopamine and that the strongest signals are located from the intermediate region to the soma, with low expression at the tip of the astrocytic process.

The spatial sequence of Ca^2+^ signals for the weak dopaminergic input (*ν*_*d*_ = 0.1 Hz) is shown in the raster plot of Fig 4A (middle plot). The signals start later than those induced by glutamate (compare with the bottom plot in the same figure). Interestingly, the second block of signals begins in the intermediate compartments and only reaches the proximal compartments and soma later. This may be due to the accumulation of Ca^2+^ and IP_3_ in the intermediate compartments after the first block, which were transported by diffusion from the soma and proximal compartments. Since the dopaminergic stimulation is weak, there was not enough time for the replenishment of Ca^2+^ and IP_3_ in the soma and proximal compartments to sufficient levels for them to initiate the next block. This phenomenon was not observed for higher frequencies of dopaminergic stimulation.

#### Interaction between local glutamatergic and global dopaminergic stimulation

Our study of the interaction between glutamate and dopamine began by combining weak stimulation by the two neurotransmitters as considered above (*ν*_*g*_ = 2 Hz and *ν*_*d*_ = 0.1 Hz) The result is shown in the raster plot of Fig 4A (top plot). The first block of Ca^2+^ signals occurs around 30 s (earlier than that induced by glutamate alone) and, as in the case of glutamate alone, starts from the first of the distal compartments and propagates to its neighbors; however, in this case it reaches compartment 9, which is the most distal of the astrocytic process. In the subsequent blocks of Ca^2+^ signals, the extent of the activated region reaches, at most, the beginning of the proximal region (compartment 4), but never extends beyond it. This behavior is different from what would be obtained by simply superimposing the raster plots for glutamate and dopamine alone, indicating that there is indeed an interaction between the two neurotransmitters.

First, dopamine enhanced the glutamatergic activation of compartment 9, facilitating the occurrence of Ca^2+^ signals in this compartment at the tip of the astrocytic process. The above study of isolated stimulations by glutamate and dopamine (cf. ‘Local Glutamatergic Input’ and ‘Global Dopaminergic Input’) showed that weak glutamate and dopamine inputs produced low-amplitude fluctuations in compartment 9 in the case of glutamate, and very weak ones in the case of dopamine. However, when a weak input of dopamine was added to a weak input of glutamate the interaction between the two allowed the occurrence of distal Ca^2+^ signals. The facilitatory effect observed for dopamine appears to be exclusive to global-acting neurotransmitters like it. Control studies in which global dopaminergic stimulation was replaced with local glutamatergic stimulation applied to the soma did not produce the same effects seen with dopamine. For further details, see the S4 Fig.

The second effect of the interaction was that glutamate inhibited the dopamine-induced activity in the soma and proximal compartments. Weak dopaminergic input alone produced activation in all compartments except the most distal ones (cf. ‘Global Dopaminergic Input’), but when it occurred together with weak glutamatergic input, activity in the soma and proximal compartments was suppressed.

A possible explanation for these effects is based on those given for the results with glutamate and dopamine stimulation alone. Although glutamate and dopamine were applied simultaneously, the response to glutamate is faster, starting in the distal and intermediate compartments. This generated small fluxes of Ca^2+^ and IP_3_ into the intermediate compartments, which, combined with the increase in the concentrations of these molecules caused by dopaminergic stimulation, resulted in the occurrence of Ca^2+^ signals in the intermediate compartments before than in the proximal compartments and the soma. This “reversal” in the order of activation between the intermediate and proximal compartments (compared to what was observed with dopamine stimulation alone) led to Ca^2+^ and IP_3_ levels in the somatic and proximal compartments that were insufficient to produce Ca^2+^ signals in these compartments. In contrast, the increase in Ca^2+^ concentration in distal compartments 8 and 9 was sufficient to generate Ca^2+^ signals in these compartments.

To further explore the interaction between glutamate and dopamine, we performed experiments with different combinations of glutamatergic and dopaminergic input frequencies and the results are summarized in the diagrams of Fig 4B. These diagrams show the frequency and amplitude of Ca^2+^ signals in compartment 9, which was the weakest activated by glutamate or dopamine alone. Dopamine stimulation lowered the frequency threshold of glutamate stimulation required to trigger Ca^2+^ signals in this compartment. The frequency threshold for glutamate input to trigger Ca^2+^ signals in the absence of dopamine (top row of the diagrams) was about 2 Hz. Introduction of a dopaminergic input at a frequency of 0.1 Hz lowered this threshold to 1 Hz, and increasing the dopamine frequency to 0.5 Hz allowed Ca^2+^ signals to be triggered even with glutamatergic input frequencies as low as 0.1 Hz. To confirm that this was a facilitatory effect of dopamine on glutamate input, thus dependent on the interaction between the two neurotransmitters, and not a consequence of dopamine alone, look at the leftmost column in these diagrams, corresponding to the case without glutamate. One sees that dopamine frequencies of 0.1 and 0.5 Hz did not elicit Ca^2+^ signals, meaning that glutamate input was necessary for generating Ca^2+^ signals in the distal compartment.

Higher glutamate input frequencies (starting from 3 Hz) could already produce Ca^2+^ signals at compartment 9, independently of dopamine, but there was still a reinforcing effect of dopamine, as higher dopaminergic input frequencies increased the frequency of Ca^2+^ signals. A symmetrical effect occurred at high dopaminergic input frequencies (e.g., above 1 Hz), where dopamine alone could generate Ca^2+^ signals in the compartment, but the frequency increased with higher glutamatergic input frequencies. For very high stimulation frequencies, this relatively symmetric interaction between glutamate and dopamine in generating Ca^2+^ signals became slightly biased in favor of glutamate. A glutamatergic input at 10 Hz could produce several Ca^2+^ signals during a stimulation trial, even with low dopaminergic input frequencies, but a dopaminergic input at 10 Hz only generated similar amounts of Ca^2+^ signals at high glutamatergic input frequencies.

### Phase-plane analysis of the simplified model

#### Influence of compartment radius

**Fig 5:**
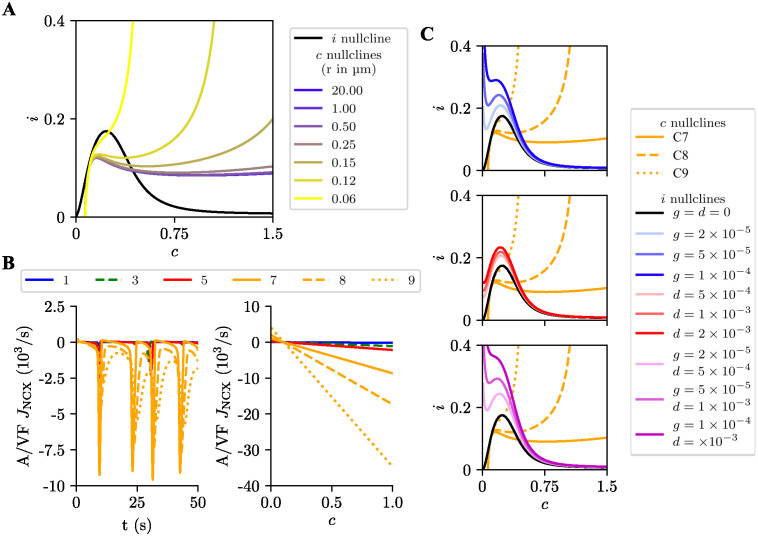
Compartmentalized dynamics in the simplified model. (**A**) (*c*-*i*) phase plane of the simplified compartment model showing the nullclines at rest (*g* = *d* = 0) for different radii (*r*). The black line is the *i* nullcline, and the yellow to blue lines (depicted in different colors) represent the *c* nullclines. (**B**) Left panel: *J*_NCX_ current density in selected compartments of the unipolar model (color-coded as indicated atop) for glutamatergic input delivered to all compartments at 10 Hz. Currents were adjusted to compartment size by the factor A/VF. Right panel: dependence of *J*_NCX_ on intracellular Ca^2+^ concentration *c*. (**C**) Nullclines for the uncoupled distal compartments (7, 8 and 9) under different types of stimulation: only glutamate input (top), only dopamine input (middle), and glutamate and dopamine inputs together (bottom). The yellow lines represent the *c* nullclines for compartments 7 (solid), 8 (dashed), and 9 (dotted). The black, blue, red and purple lines represent the *i* nullclines without input (*g* = *d* = 0) and for different values of glutamate (*g*, blue), dopamine (*d*, red) and glutamate plus dopamine (*g* + *d*, purple) inputs, respectively. The shading level represents the input strength taken as the average glutamate and dopamine values (dimensionless variables) for inputs of 2 Hz (*g* = 2 × 10^−5^; *d* = 5 × 10^−4^), 5 Hz (*g* = 5 × 10^−5^; *d* = 1 × 10^−3^), and 10 Hz (*g* = 1 × 10^−4^; *d* = 2 × 10^−3^).

Fig 5A shows the *i* and *c* nullclines of the simplified model at rest for different values of the compartment radius. The *c* nullcline depends on the compartment radius; for compartments with large radii, the *c* nullcline has a plateau-like shape, while for compartments with small radii (below ∼ 0.50 μm) it displays a sharp increase as a function of *c*. The *i* nullcline, on the other hand, does not depend on the radius; it has a right-skewed bell shape, reflecting the dependence of IP_3_ synthesis on Ca^2+^ concentration.

**Fig 6:**
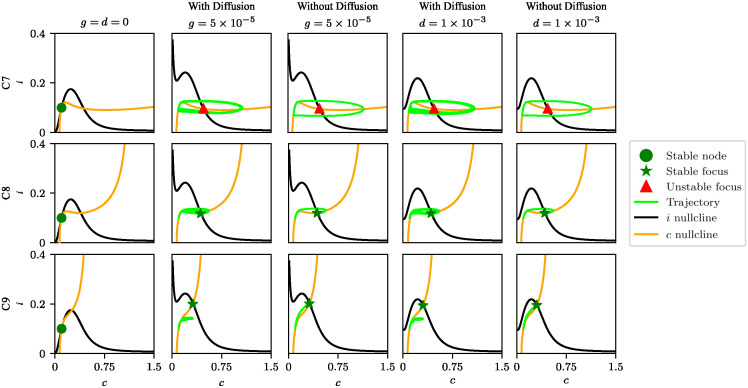
Phase portraits of the simplified model. Top row: compartment 7; middle row: compartment 8; bottom row: compartment 9. The first column corresponds to rest; the second and third columns correspond to glutamatergic input alone (*g* = 5 × 10^-5^) with and without compartmental coupling by diffusion, respectively; and the fourth and fifth columns correspond to dopaminergic input alone (*d* = 1 × 10^-3^) with and without compartmental coupling by diffusion, respectively. The yellow and black solid lines represent, respectively, the *c* and *i* nullclines, and the green line the trajectory. Green dots represent stable nodes, green stars stable foci and red triangles unstable foci.

The behavior of the nullclines as a function of compartment radius has a significant effect on compartment dynamics. To characterize this behavior, we selected the distal compartments (7, 8, and 9) as representative of the compartments in the unipolar morphology and constructed the phase portraits for these compartments (see Fig 6). As shown in the left column of this figure, at rest the system remains in its stable equilibrium state.

Moving on to the study of glutamate or dopamine stimulation, let us then start with the configuration where the compartments are uncoupled, which was implemented by setting *D*_Ca_ = 0 and $D_{{\text{IP}}_{3}}=0$ in the model equations (see columns labeled ‘Without Diff’ in Fig 6). For compartment 7, both glutamatergic and dopaminergic inputs shifted the equilibrium point to the center of the phase plane, turning it into an unstable focus; consequently, the system enters an oscillatory regime. In contrast, for compartments 8 and 9, their new equilibrium points become a stable focus and a stable node, respectively, leading their trajectories to converge to new equilibrium states. When the compartments are coupled (see columns labeled ‘With Diff’ in Fig 6), their behavior is similar to what they exhibit without coupling. However, the oscillation in compartment 8 becomes damped and the trajectory of compartment 9 converges to a fixed point near the equilibrium point of compartment 8. The explanation for this latter behavior is that the diffusion of Ca^2+^ and IP_3_ from compartment 8 to 9 drives the concentrations of these molecules in compartment 9 closer to their equilibrium values in compartment 8.

The phase plane analysis shows that thicker compartments, such as compartment 7, produce high-amplitude Ca^2+^ signals, whereas thinner compartments, such as compartment 9, merely accumulate Ca^2+^ and IP_3_ without generating Ca^2+^ signals.

To explore further the effect of compartment radius on the *c* dynamics, we studied how the compartment radius affects the NCX current density *J*_NCX_ adjusted for compartment size (see Eq 2). Simulating a glutamatergic input of 10 Hz to all compartments, the current density *J*_NCX_ was stronger in the distal compartments than in the other compartments (Fig 5B, left panel). Moreover, *J*_NCX_ has a linear dependence on *c* (Fig 5B, right panel). As *c* increases, the difference between the magnitudes of *J*_NCX_ for distal and proximal compartments also increases. Indeed, varying the parameters of maximum NCX activity and GluT currents in the detailed model affected the frequency of Ca^2+^ signals, and their amplitude and propagation (S3 Fig).

#### Influence of stimulation type

The effect of changing the strength of the input type (glutamate alone, dopamine alone, and glutamate and dopamine together) on the distal compartments is shown on the phase plane plots of Fig 5C. While the *c* nullcline is independent of input type, the *i* nullcline is much more sensitive to glutamate than to dopamine. As a consequence, increasing the strength of glutamatergic input shifts the left side of the *i* nullcline upward. This moves the equilibrium point of compartments 7 and 8 to the right and increases their equilibrium *c* value. On the other hand, it moves the equilibrium point of compartment 9 upwards to higher *i* equilibrium values (Fig 5C, top). In contrast, dopamine has a weaker effect on the dynamics, moving the *i* nullcline slightly upward and to the right (Fig 5C, middle). However, when applied together with glutamate, dopamine enhances the effect of glutamatergic input (Fig 5C, bottom). Compared to the condition with glutamate alone, dopamine further pushed the *i* nullcline upward and to the right. Hence, glutamate is more efficient in producing Ca^2+^ signals in distal compartments than dopamine. This explains the bias toward glutamate observed in the analysis of the diagrams in Fig 4B. Dopamine, when applied together with glutamate, amplified the effect of glutamate.

### Influence of morphology

#### Bipolar morphology

**Fig 7:**
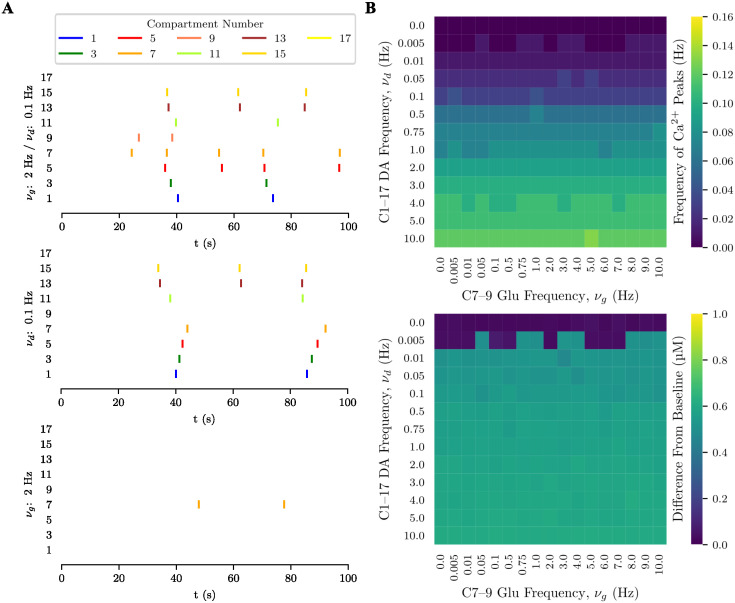
Interaction between dopaminergic and glutamatergic inputs in the bipolar morphology. (**A**) Raster plot of Ca^2+^ signals in selected compartments of the bipolar model (soma (1), lower process (3, 5, 7, and 9) and upper process (11, 13, 15 and 17)) during a 100 s trial in which the upper process received weak (1 Hz) glutamatergic input at its distal compartments for different frequency combinations of local glutamatergic input at the lower primary process and global dopaminergic input (time in horizontal axis and compartment number in the vertical axis; vertical bars—color-coded according to process region (yellow: distal; red: intermediate; green: proximal; blue: soma)—indicate Ca^2+^ signals). From top to bottom: joint glutamate and dopamine inputs with *ν*_*g*_ = 2 Hz and *ν*_*d*_ = 0.1 Hz; dopamine input only with *ν*_*d*_ = 0.1 Hz; and glutamate input only with *ν*_*g*_ = 2 Hz. (**B**) frequency (top diagram) and amplitude (bottom diagram) of Ca^2+^ peaks in compartment 15 of the upper primary process for different frequency combinations of *ν*_*g*_ and *ν*_*d*_.

In all experiments with this morphology, the distal compartments of the upper process (15, 16, and 17) received weak (1 Hz) glutamatergic input (see S1 Text for detailed stimulation protocols). A 2 Hz glutamate input to the distal compartments of the lower process (7, 8, 9) did not induce Ca^2+^ signals in the upper process (Fig 7A, bottom plot), nor did any frequency of glutamate input to the lower process elicit Ca^2+^ signals in compartment 15 of the upper process (Fig 7B, top rows). Thus, glutamatergic stimulation of one process did not propagate through the soma to the other process. Dopaminergic input at 0.1 Hz triggered Ca^2+^ signals across both processes, except in the most distal compartments (9 and 17) (Fig 7A, middle plot). In the specific study for compartment 15, dopaminergic input above 0.01 Hz was sufficient to evoke Ca^2+^ signals (Fig 7B).

Combined glutamate and dopamine stimulation had minimal impact on the upper process but facilitated responses in the lower process, even triggering Ca^2+^ signals in its distal compartment (Fig 7A, top plot). Unlike in the unipolar morphology, glutamate stimulation did not prevent dopaminergic-induced responses in proximal compartments, likely due to increased Ca^2+^ and IP_3_ fluxes reaching these processes. In compartment 15 of the upper process, responses were mainly governed by dopamine, with negligible influence from increasing glutamatergic input frequency in the distal compartments of the lower process.

The lack of communication between processes observed here can be explained considering that the response elicited in a process needs to cross the soma in order to reach the other process and influence its activity. Due to the weaker coupling strength between the soma and its neighboring compartments (see equations (S16) and (S17)), small amounts of Ca^2+^ and IP_3_ diffuse to/from the soma, restricting interaction between processes connected through it.

#### Bifurcated-terminal morphology

**Fig 8:**
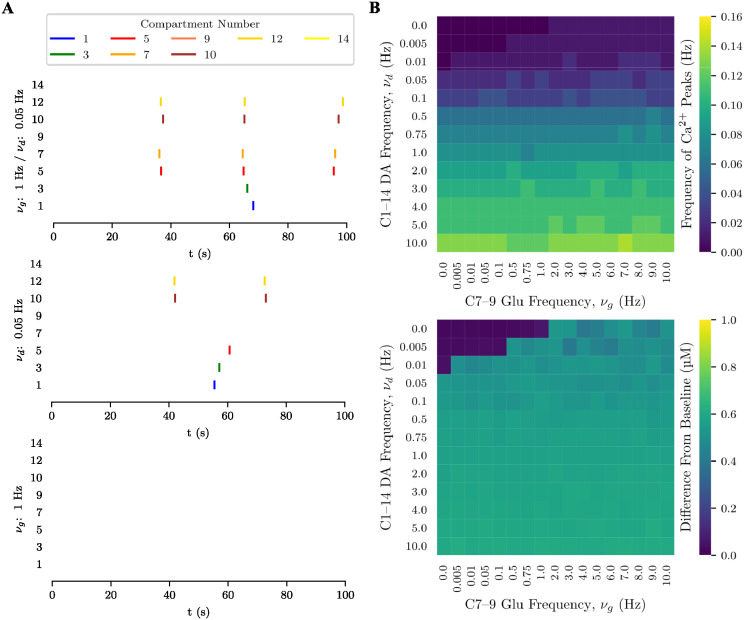
Interaction between dopaminergic and glutamatergic inputs in the bifurcated-terminal morphology. (**A**) Raster plot of Ca^2+^ signals in selected compartments of the bifurcated-terminal model (soma (1), primary process (3), lower secondary process (5, 7 and 9), and upper secondary process (10, 12 and 14)) during a 100 s stimulation trial in which the upper secondary process received weak (1 Hz) glutamatergic input for different frequency combinations of local glutamatergic input at the lower secondary process and global dopaminergic input (time in horizontal axis and compartment number in the vertical axis; vertical bars—color-coded according to process region (yellow: distal; red: intermediate; green: proximal; blue: soma)—indicate Ca^2+^ signals). From top to bottom: joint glutamate and dopamine inputs with *ν*_*g*_ = 1 Hz and *ν*_*d*_ = 0.05 Hz; dopamine input only with *ν*_*d*_ = 0.05 Hz; and glutamate input only with *ν*_*g*_ = 1 Hz. (**B**) frequency (top diagram) and amplitude (bottom diagram) of Ca^2+^ peaks in compartment 12 of the upper secondary process for different frequency combinations of *ν*_*g*_ and *ν*_*d*_.

In all experiments with this morphology, the distal compartments of the upper secondary process (12, 13, 14) received a weak (1 Hz) glutamatergic input (more details of the stimulation protocol are given in S1 Text). Weak glutamatergic input (1 Hz) in the lower secondary process did not trigger Ca^2+^ signals in the upper secondary process (Fig 8A, bottom plot). On the other hand, weak dopaminergic stimulation (0.005 Hz) activated several compartments and even a distal one in the upper secondary process (Fig 8A, middle plot). The combination of the weal glutamatergic and dopaminergic inputs enhanced the dopamine-induced response, increasing the frequency of Ca^2+^ signals (Fig 8A, top plot). Similarly to the unipolar morphology, the glutamatergic stimulation delayed the response to dopamine in the proximal and somatic compartments in comparison to the condition with dopaminergic stimulation alone.

Differently from the bipolar morphology, in the bifurcated-terminal morphology the glutamatergic stimulation of the lower secondary process evoked responses in compartment 12 (the first distal compartment from the soma) of the upper secondary process (Fig 8B). This happened for stimulation frequencies *ν*_*g*_ = 2 Hz and above. This suggests that two processes connected through a non-somatic parent process can interact with each other by glutamatergic-induced stimulation. This interaction was facilitated by dopamine, which reduced the threshold glutamatergic input frequency to 0.5 Hz for *ν*_*d*_ = 0.005 Hz and 0.005 for *ν*_*d*_ = 0.01 Hz (Fig 8B). Moreover, dopaminergic input alone could trigger responses in compartment 12 for frequencies above 0.01 Hz (Fig 8). For dopaminergic stimulation sufficiently strong (*ν*_*d*_ > 0.1 Hz), the glutamatergic influence became negligible.

Together, these results suggest that the interaction between processes depends on their hierarchical order, being weak when they are primary, i.e. directly connected to the soma, and stronger when they are secondary or of higher order, i.e. not connected through the soma. This interaction is facilitated when the sites of glutamate-induced activity are closer to the bifurcation points and weakened when they are farther apart (see S6 and S7 Figs). Moreover, they suggest that the branching pattern of the astrocytic processes has an important impact on the interaction between different regions of an astrocyte.

## Discussion

We developed a detailed compartmental model of astrocytes incorporating both glutamatergic and dopaminergic stimulation mechanisms to examine their effects on intracellular Ca^2+^ and IP_3_ dynamics. Glutamate stimulation produced localized responses with little spread beyond the stimulation sites, whereas dopamine activated a global response across the cell. The model demonstrated strong compartmentalization, with distinct activity patterns between distal and proximal compartments. Dopamine was shown to enhance glutamatergic activity by lowering the threshold frequency required to elicit Ca^2+^ responses. This enhancement resulted from the interaction between the two neurotransmitters, as neither alone, at low frequency, was able to generate Ca^2+^ signals. To investigate the underlying mechanisms of the compartmentalized dynamics, a simplified model was developed, revealing that compartment-specific responses are driven by differences in compartment radii. The simplified model further confirmed the modulatory role of dopamine, amplifying glutamatergic effects at distal compartments. Finally, analysis of branched models with varying morphologies indicated that inter-process interactions depend on their hierarchical arrangement in a dopamine-dependent manner.

The responses triggered by glutamate and dopamine in the present model are consistent with previous reports, showing similar duration, amplitude and compartmentalization compared to other computational [16, 19, 34] and experimental [20] studies. The different shapes of Ca^2+^ signals between the somatic and proximal compartments and the distal compartments can be attributed to the different Ca^2+^ and IP_3_ dynamics between these compartments, and to the neurotransmitter type, as shown by the analysis of the phase space of the simplified model for different radii. Thicker compartments have higher ER volume (higher *r*_ER_ value) and so the IP_3_-dependent mechanism is stronger in these regions, triggering high-amplitude and short-duration Ca^2+^ signals. In contrast, the NCX-dependent mechanism is stronger in thinner compartments, being responsible for Ca^2+^ signals with smaller amplitudes and longer durations. As a consequence, thinner compartments tend to accumulate IP_3_ without significant intracellular Ca^2+^ variation. This suggests that thinner processes act as sources of IP_3_ that can diffuse from the stimulated region and facilitate the response in other astrocyte regions by bringing their intracellular concentration closer to the threshold for triggering Ca^2+^ events. The phase plane analysis of the simplified model also suggested that dopamine has a modulatory role on the astrocyte activity, amplifying the response to local glutamate input.

In this sense, global responses triggered by dopaminergic stimulation modulated the activity in the distal regions of the unipolar morphology, facilitating the generation of Ca^2+^ signals in the distal compartments through the diffusion of Ca^2+^ and IP_3_. Interestingly, and in contrast with this facilitatory role of dopamine, glutamatergic input prevented or delayed the response to low frequency dopaminergic stimulation. Intracellular Ca^2+^ increases the degradation of IP_3_ by the enzyme IP_3_-3K (equation (S12)) and inhibits IP_3_ synthesis by glutamatergic and dopaminergic receptors (equations (S9) and (S10)). Since stimulating the model with glutamate promotes the diffusion of Ca^2+^ from the distal compartments, this stimulation slightly increased the Ca^2+^ concentration without triggering Ca^2+^ signals, therefore preventing responses in neighboring compartments. This effect was not observed for higher stimulation frequencies, in which case lager fluxes of Ca^2+^ and IP_3_ reduced the input threshold frequency required to trigger Ca^2+^ signals.

Since dopamine facilitated the response in the distal compartments of the unipolar morphology, we further explored this facilitatory effect of dopamine using branched astrocyte morphologies with different hierarchical organizations. No clear interaction was detected between glutamatergic inputs in the bipolar morphology, where the two first-order processes are connected through the soma. Given the larger volume of the somatic compartment, reflected in lower coupling strengths with neighboring compartments (equations (S16) and (S17)), the soma acts as a “sink” of Ca^2+^ and IP_3_. So, besides the distance between the terminal points of the two processes in the bipolar morphology, the interaction between the two depends on signal-crossing through the somatic barrier, making it difficult the communication between processes that emanate directly from the soma. In contrast, the second-order processes connected through a non-somatic parent process in the bifurcated-terminal morphology can interact by glutamate-induced activity at their tips. This interaction is dependent on the distance between the process tips, thus on their lengths. Additionally, the global dopaminergic input facilitated the communication between the secondary processes. The conclusion of these studies is that the hierarchical order of the astrocytic processes interfere in their communication.

Global responses in astrocytes can be triggered by neurotransmitters [34, 53, 54]. The number of bigger events increases with neurotransmitter input [54], and the propagation of Ca^2+^ signals between astrocytes is facilitated by sparse connectivity, but damped with the number of unactivated neighbors [57, 58]. Since the IP_3_ synthesized in the activated astrocytes diffuses and is diluted among the unactivated ones, the activated cells could be seen as IP_3_ “sources” while the unactivated as IP_3_ “sinks” [57, 58]. Interestingly, linear coupling between astrocytes also prevents IP_3_ accumulation and reduces the reach of Ca^2+^ signals propagated through the astrocytic network [54]. Compared to these studies, the compartments in the present model can be seen as nodes connected by linear diffusion and the activation of non-stimulated neighboring compartments as the propagation of Ca^2+^ signals between astrocytes [54, 57, 58]. Thus, the distal compartments would be major IP_3_ “sources” connected by linear diffusion to unactivated compartments that do not receive direct glutamatergic input, failing to activate the remaining compartments. In contrast, dopamine activates all compartments, decreasing the number of unactivated nodes and so enhancing the transmission of Ca^2+^ and IP_3_ along the astrocyte. This explains why dopamine facilitated the communication between compartments in the unipolar and bifurcated-terminal morphologies. Since the compartments are connected by linear diffusion, it prevents IP_3_ accumulation and limits the propagation range of Ca^2+^ signals in the astrocytic process [48], explaining the absence of communication between primary processes in the bipolar morphology and the limiting effect of compartment and processes length on Ca^2+^ propagation.

Our model predicts that the frequency of global events and the size of propagated Ca^2+^ waves between astrocytes increase with neuromodulator input, even when they do not trigger Ca^2+^ signals. In addition, our model predicts that secondary or higher order processes with closer bifurcation points have stronger communication and are more likely to have synchronized responses than distant processes. This synchronized response should be enhanced in the presence of neuromodulators.

Dopamine is involved in different brain functions, such as control of motor behavior, processing of stimulus salience, and gating of information in working memory [24–27]. Astrocytes regulate neural oscillations in the prefrontal cortex and hippocampus [10, 11], regions that are synchronized during working memory tasks [59, 60]. Since gliotransmitters can modulate synaptic activity, enhancing the communication between two neurons [34], neuromodulators could influence several synapses at the same time by triggering global events and promoting simultaneous release of gliotransmitters at several locations, linking relevant behaviors or salient stimuli to the modulation of wide neuron populations [1, 5, 8, 23]. As dopaminergic transmission in prefrontal cortex is associated with modulation of oscillatory activity [41], it is possible that dopamine regulates the oscillatory activity and working memory by triggering global events, enhancing the communication between astrocytic processes, and so synchronizing synaptic activity. Reduced dopaminergic transmission and its effect on astrocytes in the prefrontal cortex could then be the basis for the working memory deficits detected in some psychiatric conditions with lower dopaminergic drive in this region, such as schizophrenia and attention-deficit/hyperactivity disorder [27, 32]. Similar to dopamine, other neuromodulators could also be associated with global responses in astrocytes and modulation of large neural ensembles. Noradrenaline, for example, triggers broad responses in astrocytes and is associated with neural synchronizations [3, 55, 56]. Our model predicts that neural synchronization modulated by astrocytes is stronger for neurons contacted by branches that have closer branching point and that this synchronization should be enhanced by neuromodulators, such as dopamine and noradrenaline, being correlated with the occurrence of global events.

## Conclusion

Previous computational studies showed that astrocytes integrate inputs from distinct synapses and trigger global responses when these inputs are synchronized [34, 53]. Other approaches showed that these broad responses can also emerge from spontaneous synchronized responses and by intrinsic mechanisms of the IP_3_ dynamics [54]. Here we expanded these studies using compartmental models of astrocytes and implementing mechanisms related to ionic transport across the cell membrane [19] and dopaminergic transmission [18, 29], investigating the interplay between local and global responses and how morphological characteristics of the astrocyte could influence the Ca^2+^ response. Global events, prompted by neuromodulators, may facilitate communication between astrocytic processes that would otherwise operate independently. This interconnection can improve the response to low-frequency synaptic input and enable simultaneous modulation of multiple synapses. By comparing three different morphologies, we observed distinct modulatory effects, underscoring the dependence of these effects on spatial characteristics. In addition to the detailed model, the simplified model presented here captures well the behavior of intracellular Ca^2+^ and IP_3_ dynamics at a relatively low computational cost, allowing the construction of large-scale astrocytic networks. Since it is a two-dimensional model, its behavior can be studied by analytical tools to deepen our understanding of dynamic mechanisms. Further studies are required to validate the model predictions presented here and to elucidate the impact of dopaminergic transmission on astrocyte activity.

## Supporting information

S1 Text Supplementary material. Supplementary text with model description, stimulation protocols, control tests, computational and numerical methods, and model limitations.
(PDF)

S1 Table Model parameters. Tables listing all parameters of the detailed and simplified models.
(PDF)

S1 Fig Unipolar model response to glutamate input at different stimulation sites. Model response to glutamatergic stimulation of the (**A**) somatic compartment (C1), (**B**) proximal compartment (C3) and (**C**) intermediate compartment (C5), at frequencies *ν*_*g*_ of 5 or 10 Hz. The resulting Ca^2+^ signals propagate from the stimulation site to neighboring compartments, but no single input fully activates all astrocytic compartments.
(EPS)

S2 Fig Simplified unipolar model response to glutamate and dopamine input. (**A**) Glutamate stimulation of distal compartments (7, 8, and 9) elicits Ca^2+^ signals in distal and intermediate compartments at input frequencies above 1 Hz. (**B**) Whole-cell dopamine input triggers Ca^2+^ signals across all compartments. Enlarged time plots provide details of the Ca^2+^ signals. The Ca^2+^ signals generated in the simplified model closely resemble those observed in the detailed model.
(EPS)

S3 Fig Effect of GluT and NCX currents on Ca^2+^ signals in distal compartments. (**A**) [Ca^2+^]_*i*_ time series for three values of *J*_NCXmax_ (top: 0; middle 0.0001 pA/μm^2^; bottom: 0.001 pA/μm^2^). Glutamatergic input was applied to compartment 9 at a frequency of 10 Hz for 60 s. With *J*_NCXmax_ = 0, the amplitude of [Ca^2+^]*i* and the number of Ca^2+^ signals triggered in distal compartments increased compared to conditions with higher *J*NCXmax values. Thus, higher *J*_NCXmax_ values reduce the number and amplitude of Ca^2+^ signals, indicating that NCX currents attenuate the effects of glutamatergic stimulation on [Ca^2+^]*i* and its propagation. (B) Ca^2+^ signal amplitudes in compartment 8 for different *J*NCXmax and *J*_GluTmax_ values. (C) Ca^2+^ signal amplitudes in compartment 9 for different *J*_NCXmax_ and *J*_GluTmax_ values. In compartment 8, the NCX current is the primary regulator of [Ca^2+^]*i*, as changes in *J*GluTmax did not affect [Ca^2+^]*i* amplitude. In contrast, in compartment 9, increasing *J*GluTmax led to a slight increase in [Ca^2+^]_*i*_ amplitude.
(EPS)

S4 Fig Interaction between somatic and distal glutamatergic stimulation. Frequency (left) and amplitude (right) of Ca^2+^ signals in (**A**) compartment 9 of the unipolar morphology, (**B**) compartment 15 of the bipolar morphology, and (**C**) compartment 14 of the bifurcated-terminal morphology under combined somatic and distal compartment glutamatergic stimulation.
(EPS)

S5 FigCa^2+^ and IP_3_ diffusion control the propagation range of Ca^2+^ signals.(**A**) frequency of Ca^2+^ signals triggered with glutamate input to compartment 2 (*ν*_*g*_: 1 Hz) and distal compartments 7, 8 and 9 (*ν*_*g*_: 0.5 to 10 Hz) in the unipolar morphology. The vertical axis gives the values of the diffusion coefficients *D*_Ca_ and $D_{{\text{IP}}_{3}}$. (**B**) Ca^2+^ signal propagation range for various diffusion coefficients (horizontal axis) in the original unipolar morphology (9 comparts.) and in extended versions (10 and 11 comparts.). The propagation range is defined as the number of activated compartments counted from the most distal compartment. The additional compartments in the extended morphologies match the radius of compartment 5 (10-compartment model) and compartments 5 and 7 (11-compartment model).(EPS)

S6 Fig The interaction between dopamine and glutamate responses depends on the bifurcation point. Frequency of Ca^2+^ signals for various frequency combinations of local glutamate and global dopamine inputs. Compartment lengths and radii are the same as in the original bifurcated-terminal morphology. The upper secondary process received low-frequency glutamatergic input (*ν*_*g*_: 1 Hz), while different frequency combinations of local glutamate were applied at distal compartments in the lower secondary process, along with global dopamine input.
(EPS)

S7 Fig The facilitatory effect of dopamine depends on the length of the secondary processes. Frequency of Ca^2+^ signals for different frequency combinations of local glutamate and global dopamine. Radii (Φ) of the additional compartments are indicated in the schematic representation of the morphological model; all additional compartments have a unit length. The upper and longer secondary process received low-frequency glutamatergic input (*ν*_*g*_: 1 Hz), with different local glutamate frequency combinations at the distal compartments of the lower secondary process, along with global dopamine input.
(EPS)
