# Supplementary material for: Dopamine facilitates the response to glutamatergic inputs in astrocyte cell models
Source: PLoS Comput Biol. 2024 Dec 16;20(12):e1012688. doi: 10.1371/journal.pcbi.1012688 (PMC11684655; doi:10.1371/journal.pcbi.1012688)
Supplement: S1 Text — Supplementary text with model description, stimulation protocols, control tests, computational and numerical methods, and model limitations. (PDF) [file pcbi.1012688.s001.pdf]

# Dopamine facilitates the response to glutamatergic inputs in astrocyte cell models
# S1 Text: Supplementary Material

Thiago Ohno Bezerra, Antonio C. Roque

## Detailed Model

For convenience, the units of the current densities $J_{\mathrm{NCX}}$, $J_{\mathrm{NKA}}$, $J_{\mathrm{GluT}}$, $J_{\mathrm{IP_3R}}$, $J_{\mathrm{SERCA}}$, $J_{\mathrm{CaERL}}$, $J_{\mathrm{NaL}}$, $J_{\mathrm{KL}}$ were chosen as pA/µm$^2$. This was done to simplify the calculation of the compartment potential $v$ (equation (9) of the main text). To calculate the rates of change of molar concentrations (in µM/s), current densities were multiplied by the factor $(A/VF)$, where $F$ is the Faraday constant ($F$ = 96,500 C/mol), and $V$ and $A$ are the volume and area of the compartment, respectively. The biophysical interpretation of all equations and parameters can be found in [1–4]. In the following, we use the Hill function notation,

$$\mathcal{H}_n(x,k) \coloneqq x^n/(x^n+k^n),$$

which simplifies the equations. The parameter values are given after each equation below and, for convenience, also listed in Table 1 of the Supplementary Information S2 file.

The $Ca^{2+}$ current density through ER due to activation of $IP_3$ receptors is given by the following equations:

$$J_{\mathrm{IP_3R}} = \frac{FV}{A} r_C \left(\mathcal{H}_1\left([\mathrm{IP_3}], d_1\right)\right)^3 \left(\mathcal{H}_1\left([\mathrm{Ca}^{2+}]_i, d_5\right)\right)^3 h^3([\mathrm{Ca}^{2+}]_{\mathrm{ER}} - [\mathrm{Ca}^{2+}]_i), \tag{S1}$$

where $r_C = 6$ s$^{-1}$ is the maximum rate of the $Ca^{2+}$ current through the $IP_3R$ channel, $d_1 = 0.13$ µM and $d_5 = 0.08234$ µM are, respectively, the dissociation constants of $IP_3$ and $Ca^{2+}$, and $h$ represents the proportion of open $IP_3R$ channels.

The $Ca^{2+}$ leak from ER and the SERCA pump of $Ca^{2+}$ into ER current densities are described by the equations:

$$J_{\mathrm{CaERL}} = \frac{FV}{A} r_L([\mathrm{Ca}^{2+}]_{\mathrm{ER}} - [\mathrm{Ca}^{2+}]_i) \tag{S2}$$

$$J_{\mathrm{SERCA}} = \frac{FV}{A} v_{\mathrm{ER}} \mathcal{H}_2\left([\mathrm{Ca}^{2+}]_i, K_{\mathrm{ER}}\right), \tag{S3}$$

where $r_L = 0.11$ s$^{-1}$ is the $Ca^{2+}$ leak rate constant, $v_{ER} = 11.93$ µM/s is the maximum $Ca^{2+}$ uptake rate by SERCA and $K_{ER} = 0.1$ µM is the $Ca^{2+}$ affinity for the SERCA pump.

The ionic current density through GluT is governed by the equation:

$$J_{\mathrm{GluT}} = J_{\mathrm{GluTmax}} \mathcal{H}_1\left([\mathrm{K}^+]_i, K_{\mathrm{GluTmK}}\right) \mathcal{H}_3\left([\mathrm{Na}^+]_i, K_{\mathrm{GluTmN}}\right) \mathcal{H}_1\left([\mathrm{Glu}], K_{\mathrm{GluTmg}}\right), \tag{S4}$$

where $J_{\mathrm{GluTmax}} = 0.68$ pA/µm$^2$ is the maximum transport rate and $K_{\mathrm{GluTmK}} = 5{,}000$ µM/m$^2$, $K_{\mathrm{GluTmN}} = 15{,}000$ µM/m$^2$ and $K_{\mathrm{GluTmg}} = 34$ µM/m$^2$ are the half-saturation constants of $K^+$, $Na^+$ and glutamate, respectively.

The transport of $Na^+$ and $K^+$ by the $Na^+$/$K^+$-ATPase pump is modeled as:

$$J_{\mathrm{NKA}} = J_{\mathrm{NKAmax}} \mathcal{H}_{1.5}\left([\mathrm{Na}^+]_i, K_{\mathrm{NKAmN}}\right) \mathcal{H}_1\left([\mathrm{K}^+]_e, K_{\mathrm{NKAmK}}\right), \tag{S5}$$

where the maximum pump activity is $J_{\mathrm{NKAmax}} = 1.52$ pA/µm$^2$, $K_{\mathrm{NKAmN}} = 10{,}000$ µM is the half-saturation constant of $Na^+$ and $K_{\mathrm{NKAmK}} = 1{,}500$ µM is the half-saturation constant of $K^+$.

The activity of the $Na^+$/$Ca^{2+}$-exchanger is given by the equation:

$$J_{\mathrm{NCX}} = J_{\mathrm{NCXmax}} \mathcal{H}_3\left([\mathrm{Na}^+]_e, K_{\mathrm{NCXmN}}\right) \mathcal{H}_1\left([\mathrm{Ca}^{2+}]_e, K_{\mathrm{NCXmC}}\right) \cdot \frac{\frac{[\mathrm{Na}^+]_i^3}{[\mathrm{Na}^+]_e^3} \exp\left(\eta \frac{vF}{RT}\right) - \frac{[\mathrm{Ca}^{2+}]_i}{[\mathrm{Ca}^{2+}]_e} \exp\left((\eta - 1)\frac{vF}{RT}\right)}{1 + k_{\mathrm{sat}} \exp\left((\eta - 1)\frac{vF}{RT}\right)}, \tag{S6}$$

where $J_{\mathrm{NCXmax}} = 0.0001$ pA/µm$^2$ is the maximum NCX activity, $K_{\mathrm{NCXmN}} = 87{,}500$ µM and $K_{\mathrm{NCXmC}} = 1{,}380$ µM are, respectively, the half-saturation constants of $Na^+$ and $Ca^{2+}$, $\eta = 0.35$ is the energy barrier that controls the voltage dependence of NCX, $R = 8.314$ J/molK is the gas constant, $T = 303.16$ K is the temperature and $k_{\mathrm{sat}} = 0.1$ is the saturation constant. We fixed $\mathrm{I}_{\mathrm{NCX}_{\mathrm{max}}} = 0.0001$ pA/µm$^2$ to ensure that the glutamatergic stimulation of the distal compartments triggers $Ca^{2+}$ signals in the neighboring compartments (S5 Figure.).

The $Na^+$ and $K^+$ leak current densities are governed by the following equations:

$$J_{\mathrm{NaL}} = g_{\mathrm{NaL}}(v - E_{\mathrm{Na}}) \tag{S7}$$

$$J_{\mathrm{KL}} = g_{\mathrm{KL}}(v - E_{\mathrm{K}}), \tag{S8}$$

where $g_{\mathrm{NaL}} = 13.483$ S/m$^2$ and $g_{\mathrm{KL}} = 145.814$ S/m$^2$ are the $Na^+$ and $K^+$ conductances, respectively, and $E_{\mathrm{Na}} = 61$ mV and $E_{\mathrm{K}} = -94$ mV are their corresponding reversal potentials.

The synthesis of $IP_3$ by the activation of mGluR is related to the extracellular glutamate concentration by the equation [2, 4]:

$$S_{\mathrm{PLC}\beta_{\mathrm{Glu}}} = v_\beta \mathcal{H}_\alpha\left([\mathrm{Glu}], K_R + K_p \frac{[\mathrm{Ca}^{2+}]_i}{[\mathrm{Ca}^{2+}]_i + K_\pi}\right), \tag{S9}$$

where $v_\beta = 0.674$ µM/s is the maximum rate of $IP_3$ synthesis by PLCβ, [Glu] is the extracellular glutamate concentration (equation (12)), $\alpha = 0.7$, $K_R = 1.3$ µM is the affinity of glutamate to mGluR, $K_p = 10$ µM is the $Ca^{2+}$-dependent inhibition of PLC and $K_\pi = 0.6$ µM is the affinity constant of $Ca^{2+}$ to PLC.

The synthesis of $IP_3$ by the activation of $D_1/\alpha_1$ receptors [5, 9] is modeled as:

$$S_{\mathrm{PLC}\beta_{\mathrm{DA}}} = v_{\mathrm{DA}} \mathcal{H}_\beta\left([\mathrm{DA}], K_{\mathrm{DA}} + K_p \frac{[\mathrm{Ca}^{2+}]_i}{[\mathrm{Ca}^{2+}]_i + K_\pi}\right), \tag{S10}$$

where $v_{\mathrm{DA}} = 0.025$ µM/s is the maximum production rate of $IP_3$ by PLC, [DA] is the extracellular dopamine concentration (equation (13)), $\beta = 0.5$, and $K_{\mathrm{DA}} = 5$ µM is the affinity of dopamine to the dopaminergic receptors. Since the $D_1/\alpha_1$ receptors influence the intracellular $Ca^{2+}$ concentration by activating the $IP_3$ pathway [5, 9], the dynamics of $IP_3$ production by dopamine was modeled as the

glutamate-dependent $IP_3$ synthesis [2]. We focused here on the dopaminergic pathway most studied in the literature. There are other mechanisms through which dopamine can affect the intracellular calcium concentration. Further details on these mechanisms are provided in the section on model limitations below. The values of the parameters in equation (S10) were adjusted to reproduce the amplitude and duration of the $Ca^{2+}$ signals reported in [5].

The production of $IP_3$ by PLCδ is given by [2, 4]:

$$S_{\mathrm{PLC}\delta} = \frac{v_\delta}{1 + \frac{[\mathrm{IP_3}]}{\kappa_\delta}} \mathcal{H}_2\left([\mathrm{Ca}^{2+}]_i, K_{\mathrm{PLC}\delta}\right), \tag{S11}$$

where $v_\delta = 0.025$ µM/s is the maximum rate of $IP_3$ production by PLCδ, $\kappa_\delta = 1.5$ µM is the inhibition constant of PLCδ and $K_{\mathrm{PLC}\delta} = 0.1$ µM is the $Ca^{2+}$-dependent inhibition of PLCδ.

The degradation rates of $IP_3$ by $IP_3$-3K and IP-5P are modeled according to the equations [2,4]:

$$D_{\mathrm{IP_3-3K}} = v_{3K} \mathcal{H}_4\left([\mathrm{Ca}^{2+}]_i, K_D\right) \mathcal{H}_1\left([\mathrm{IP_3}], K_3\right) \tag{S12}$$

$$D_{\mathrm{IP-5P}} = r_{5P}[\mathrm{IP_3}], \tag{S13}$$

where $v_{3K} = 2$ µM is the maximum degradation rate of $IP_3$ by $IP_3$-3K, $K_D = 0.7$ µM is the affinity of $Ca^{2+}$ to $IP_3$-3K, $K_3 = 1$ µM is the affinity of $IP_3$ to $IP_3$-3K and $r_{5P} = 0.04$ $s^{-1}$ is the maximum degradation rate of $IP_3$ by IP-5P.

# Compartmental Coupling

The diffusive flux of ion/molecule X from compartment $k$ to compartment $j$ changes the concentration of X in compartment $j$ in the following way [21]:

$$\left(\frac{d[\mathrm{X}]_j}{dt}\right)_{\mathrm{diff}} = \frac{D_\mathrm{X}\mathcal{A}_j}{V_j d_{jk}}\left([\mathrm{X}]_k - [\mathrm{X}]_j\right) = F_{\mathrm{X},k\to j}\left([\mathrm{X}]_k - [\mathrm{X}]_j\right), \tag{S14}$$

where $D_\mathrm{X}$ is the diffusion coefficient of the ion/molecule X, $\mathcal{A}_j$ is the area of the cross-section of compartment $j$, $V_j$ is the volume of compartment $j$, and $d_{jk}$ is the distance between the centers of compartments $j$ and $k$. $F_{\mathrm{X},k\to j}$ is the coupling strength for X from compartment $k$ to compartment $j$ (or simply from $k$ to $j$), and a dimensional analysis reveals that it has dimensions of $s^{-1}$.

When both compartments $j$ and $k$ are cylindrical, they have the same length $\ell$, which implies that:

$$F_{\mathrm{X},k\to j} \equiv F_{\mathrm{X,cyl}} = \frac{D_\mathrm{X}}{\ell^2}. \tag{S15}$$

Since $\ell = 1$ µm in our model, $F_{\mathrm{X,cyl}} = n\left(D_\mathrm{X}\right)$ $s^{-1}$, where $n(.)$ stands for the numerical value of a quantity without its dimensions. When compartment $k$ is the soma and compartment $j$ is a cylinder, we have:

$$F_{\mathrm{X},k\to j} \equiv F_{\mathrm{X,s\to c}} = \frac{D_\mathrm{X}}{\ell\left(r_s + \ell/2\right)}, \tag{S16}$$

where $r_s$ is the radius of soma. Since $r_s = 20$ µm in our model, $F_{\mathrm{X},s\to c} = 0.049n\left(D_\mathrm{X}\right)$ $s^{-1}$. And when compartment $k$ is a cylinder and compartment $j$ is the soma, we obtain (assuming $\mathcal{A}_j$ as the

cross-sectional area of the cylinder):

$$F_{\mathrm{X},k\to j} \equiv F_{\mathrm{X,c\to s}} = \frac{D_{\mathrm{X}} r_c^2}{\frac{4}{3} r_s^3 \left(r_s + \frac{\ell}{2}\right)}, \tag{S17}$$

where $r_c$ is the radius of the cylindrical compartment. Since in our model all cylindrical compartments connected to the soma have the same radius $r_c = 2$ µm, $F_{\mathrm{X},c\to s} = 0.183 \times 10^{-4} n\left(D_{\mathrm{X}}\right)$ s$^{-1}$.

# Simplified Model

During a 100 s stimulation trial, the behavior of the fast variables $[\mathrm{Na}^+]_i$, $[\mathrm{Na}^+]_e$, $[\mathrm{K}^+]_i$, $[\mathrm{K}^+]_e$, $v$, $[\mathrm{Ca}^{2+}]_e$ consists of random pulses, which on this time scale appear as structureless lines, interspersed with periods where they remain at their resting values. Because of this, on the time scale of the slow variables ($[\mathrm{Ca}^{2+}]_i$ and $[\mathrm{IP}_3]$), the fast variables can be approximated as remaining at their resting values (given in Table 1 of Supplementary Information S2 file) for the duration of a stimulation trial.

The variable $h$ does not change significantly during a stimulation trial, so we approximated it by its average value at glutamatergic and dopaminergic inputs of 5 Hz (see Table 2 of Supplementary Information S2 file). Next, we rescaled the intracellular $\mathrm{Ca}^{2+}$ and $\mathrm{IP}_3$ concentrations to make their resting values equal to 0.1. This was done by introducing dimensionless variables $c = \lambda[\mathrm{Ca}^{2+}]_i$ and $i = \eta[\mathrm{IP}_3]$ such that $c_{\mathrm{rest}} = i_{\mathrm{rest}} = 0.1$. Using the resting values of $[\mathrm{Ca}^{2+}]_i$ and $[\mathrm{IP}_3]$ given in Table 2 of Supplementary Information S2 file, the rescaling factors were set to $\lambda = 1.37$ mM$^{-1}$ and $\eta = 0.522$ mM$^{-1}$. With this rescaling, the dimensionless variable $c$ has its values restricted to the interval [0, 1.5]. The extracellular concentrations of glutamate and dopamine were also rescaled by their average values at a stimulus frequency of 5 Hz, resulting in the dimensionless variables $g$ and $d$. We used the average value for this rescaling because the same neurotransmission model was used for all compartments. After rescaling, all model variables, except the rate parameters (with units of s$^{-1}$) and $\alpha_{\mathrm{NCX}}$ and $\beta_{\mathrm{NCX}}$, become dimensionless. The parameters associated with the variable $c$ were scaled by the factor $\lambda$, and those associated with $i$ were scaled by the factor $\eta$, becoming dimensionless. Furthermore, the parameters in the simplified model were also adjusted to match the responses observed in the detailed model (S4 Figure). All parameters of the simplified model are given in Table 2 of the Supplementary Information S2 file.

With the concentrations of $\mathrm{Na}^+$ and $\mathrm{K}^+$ fixed, only the current through NCX is maintained after the above approximations. Denoting the resting value of variable $x$ as $\overline{x}$, the current density through NCX can be simplified as follows:

$$\begin{aligned} J_{\mathrm{NCX}} =& J_{\mathrm{NCXmax}} \frac{\overline{[\mathrm{Na}^+]_e}^3}{\overline{[\mathrm{Na}^+]_e}^3 + K_{\mathrm{NCXmN}}^3} \frac{\overline{[\mathrm{Ca}^{2+}]_e}}{\overline{[\mathrm{Ca}^{2+}]_e} + K_{\mathrm{NCXmC}}} \cdot \\ & \frac{\frac{\overline{[\mathrm{Na}^+]_i}^3}{\overline{[\mathrm{Na}^+]_e}^3} \exp\left(\eta \frac{\overline{v}F}{RT}\right) - \frac{[\mathrm{Ca}^{2+}]_i}{\overline{[\mathrm{Ca}^{2+}]_e}} \exp\left((\eta-1)\frac{\overline{v}F}{RT}\right)}{1 + k_{\mathrm{sat}} \exp\left((\eta-1)\frac{\overline{v}F}{RT}\right)} \\ =& \beta_{\mathrm{NCX}} - \alpha_{\mathrm{NCX}} c \end{aligned} \tag{S18}$$

where $\alpha_{\mathrm{NCX}} = 1.169\times10^{-4}$ A/mM.m$^2$ and $\beta_{\mathrm{NCX}} = 1.232\times10^{-5}$ A/mM.m$^2$. We arrived at these parameter values after variable rescaling as described above.

To eliminate the differential equation for $[\mathrm{Ca}^{2+}]_{\mathrm{ER}}$ we first note that the equations (2) and (4) of the main text can be combined to give:

$$\frac{d[\mathrm{Ca}^{2+}]_i}{dt} = \frac{A}{VF} J_{\mathrm{NCX}} - r_{\mathrm{ER}} \frac{d[\mathrm{Ca}^{2+}]_{\mathrm{ER}}}{dt}. \tag{S19}$$

Assuming that $J_{\mathrm{NCX}} \approx 0$ we get:

$$\frac{d([\mathrm{Ca}^{2+}]_i + r_{\mathrm{ER}}[\mathrm{Ca}^{2+}]_{\mathrm{ER}})}{dt} = 0,$$

which implies that

$$[\mathrm{Ca}^{2+}]_i + r_{\mathrm{ER}}[\mathrm{Ca}^{2+}]_{\mathrm{ER}} = \beta''_{\mathrm{CaER}},$$

where $\beta''_{\mathrm{CaER}}$ is a constant. Therefore, $[\mathrm{Ca}^{2+}]_{\mathrm{ER}}$ can be written as:

$$[\mathrm{Ca}^{2+}]_{\mathrm{ER}} = \beta'_{\mathrm{CaER}} - \frac{1}{r_{\mathrm{ER}}}[\mathrm{Ca}^{2+}]_i, \tag{S20}$$

where $\beta'_{\mathrm{CaER}} = \beta''_{\mathrm{CaER}}/r_{\mathrm{ER}}$.

Equation (S20) gives a reasonable approximation for $[\mathrm{Ca}^{2+}]_{\mathrm{ER}}$ as a function of $[\mathrm{Ca}^{2+}]_i$. To improve the approximation, we assumed a general relationship as:

$$[\mathrm{Ca}^{2+}]_{\mathrm{ER}} = \beta'_{\mathrm{CaER}}(r_{\mathrm{ER}}) - \frac{\alpha'_{\mathrm{CaER}}(r_{\mathrm{ER}})}{r_{\mathrm{ER}}}[\mathrm{Ca}^{2+}]_i, \tag{S21}$$

where $\beta'_{\mathrm{CaER}}(r_{\mathrm{ER}})$ and $\alpha'_{\mathrm{CaER}}(r_{\mathrm{ER}})$ are functions of $r_{\mathrm{ER}}$ that were fitted based on simulations of the detailed model. The best-fitting functions were

$$\alpha'_{\mathrm{CaER}} = a_\alpha r_{\mathrm{ER}}^{-1} + b_\alpha \tag{S22}$$

and

$$\beta'_{\mathrm{CaER}} = a_\beta r_{\mathrm{ER}}^4 + b_\beta r_{\mathrm{ER}}^3 + c_\beta r_{\mathrm{ER}}^2 + d_\beta r_{\mathrm{ER}} + e_\beta, \tag{S23}$$

with $a_\alpha = -1.030 \times 10^{-4}$, $b_\alpha = 1.000$, $a_\beta = -3.579 \times 10^5$, $b_\beta = 1.519 \times 10^5$, $c_\beta = -2.190 \times 10^4$, $d_\beta = 1.222 \times 10^3$, and $e_\beta = 1.904 \times 10$. We calculated these parameters by minimizing the mean squared error between the full-model and approximated time courses of $[\mathrm{Ca}^{2+}]_{\mathrm{ER}}$ during glutamatergic and dopaminergic stimulation at 5 Hz ($\nu_g = \nu_d = 5$ Hz). Finally, writing $[\mathrm{Ca}^{2+}]_i$ in terms of the dimensionless variable $c$, and introducing the dimensionless variable $c_{\mathrm{ER}} = \gamma_{\mathrm{CaER}}(r_{\mathrm{ER}})[\mathrm{Ca}^{2+}]_{\mathrm{ER}}$ equation (S21) becomes:

$$c_{\mathrm{ER}} = \beta_{\mathrm{CaER}}(r_{\mathrm{ER}}) - \alpha_{\mathrm{CaER}}(r_{\mathrm{ER}})c, \tag{S24}$$

where

$$\beta_{\mathrm{CaER}}(r_{\mathrm{ER}}) = \gamma_{\mathrm{CaER}}(r_{\mathrm{ER}})\beta'_{\mathrm{CaER}}(r_{\mathrm{ER}}) \tag{S25}$$

and

$$\alpha_{\mathrm{CaER}}(r_{\mathrm{ER}}) = \frac{\gamma_{\mathrm{CaER}}(r_{\mathrm{ER}})\alpha'_{\mathrm{CaER}}(r_{\mathrm{ER}})}{\lambda r_{\mathrm{ER}}}. \tag{S26}$$

A dependence of the $[\mathrm{Ca}^{2+}]_{\mathrm{ER}}$ scaling factor, $\gamma_{\mathrm{CaER}}$, on the compartment radius $r_{\mathrm{ER}}$ was introduced to ensure that the simplified model remains in equilibrium when $c$ is at its resting value. A distinct value of $\gamma_{\mathrm{CaER}}(r_{\mathrm{ER}})$ was computed for each compartment using the bisection method, such that $dc/dt = 0$ at rest.

The dimensionless extracellular concentrations of glutamate, $g$, and dopamine, $d$, were described as in the detailed model (see main text):

$$\frac{\mathrm{d}g}{dt} = -G_{\mathrm{g}}g + \rho_{\mathrm{g}}\delta(t - t_{\mathrm{spike}}), \tag{S27}$$

$$\frac{\mathrm{d}d}{dt} = -G_{\mathrm{d}}d + \rho_{\mathrm{d}}\delta(t - t_{\mathrm{spike}}), \tag{S28}$$

where the presynaptic spike times $t_{\mathrm{spike}}$ are modeled as Poisson processes. The values of the parameters $G_{\mathrm{g}}$, $G_{\mathrm{d}}$, $\rho_{\mathrm{g}}$, and $\rho_{\mathrm{d}}$ are given in Table 2 of the Supplementary Information S2 file.

For the construction of the $(c, i)$ phase-plane, we used constant extracellular glutamate and dopamine concentrations, $g$ and $d$, respectively, which were treated as parameters of the model. They were taken as the average values of $g(t)$ and $d(t)$ during stimulations of 5 Hz. The final set of equations for the variable $c$ are:

$$\frac{\mathrm{d}c}{\mathrm{d}t} = \frac{A}{FV}J_{\mathrm{NCX}} + \frac{A\sqrt{\mathrm{r}_{\mathrm{ER}}}}{FV}\left(J_{\mathrm{IP_3R}} - J_{\mathrm{SERCA}} + J_{\mathrm{CaERL}}\right), \tag{S29}$$

$$J_{\mathrm{NCX}} = \beta_{\mathrm{NCX}} - \alpha_{\mathrm{NCX}}c, \tag{S30}$$

$$J_{\mathrm{IP_3R}} = \frac{FV}{A}r_C\left(\mathcal{H}_1(i, d_1)\right)^3\left(\mathcal{H}_1(c, d_5)\right)^3 h^3(c_{\mathrm{ER}} - c), \tag{S31}$$

$$J_{\mathrm{CaERL}} = \frac{FV}{A}r_L(c_{\mathrm{ER}} - c), \tag{S32}$$

$$J_{\mathrm{SERCA}} = \frac{FV}{A}v_{\mathrm{ER}}\mathcal{H}_2\left(c, K_{\mathrm{ER}}\right), \tag{S33}$$

where $r_C = 6$ s$^{-1}$, $d_1 = 0.0646$, $d_5 = 0.1074$, $h = 0.8$, $r_L = 0.11$ s$^{-1}$, and $v_{\mathrm{ER}} = 18.782$ s$^{-1}$. The equations for the variable $i$ are:

$$\frac{\mathrm{d}i}{\mathrm{d}t} = S_{\mathrm{PLC}\beta_{\mathrm{Glu}}} + S_{\mathrm{PLC}\beta_{\mathrm{DA}}} + S_{\mathrm{PLC}\delta} - D_{\mathrm{IP_3-3K}} - D_{\mathrm{IP-5P}}, \tag{S34}$$

$$S_{\mathrm{PLC}\beta_{\mathrm{Glu}}} = v_\beta\mathcal{H}_\alpha\left(g, K_R + K_p\frac{c}{c + K_\pi}\right), \tag{S35}$$

$$S_{\mathrm{PLC}\beta_{\mathrm{DA}}} = v_{\mathrm{DA}}\mathcal{H}_\beta\left(d, K_{\mathrm{DA}} + K_p\frac{c}{c + K_\pi}\right), \tag{S36}$$

$$S_{\mathrm{PLC}\delta} = \frac{v_\delta}{1 + \frac{i}{\kappa_\delta}}\mathcal{H}_2\left(c, K_{\mathrm{PLC}\delta}\right), \tag{S37}$$

$$D_{\mathrm{IP_3-3K}} = v_{3K}\mathcal{H}_4\left(c, K_D\right)\mathcal{H}_1\left(i, K_3\right),$$

$$D_{\mathrm{IP-5P}} = r_{5P}i, \tag{S38}$$

where $v_\beta = 0.211$ s$^{-1}$, $K_R = 0.104 \cdot 10^{-2}$, $K_p = 0.080$, $K_\pi = 0.821$, $v_{\mathrm{DA}} = 0.013$ s$^{-1}$, $K_{\mathrm{DA}} = 5 \cdot 10^{-3}$, $v_\delta = 0.013$ s$^{-1}$, $\kappa_\delta = 0.782$, $K_{\mathrm{PLC}\delta} = 0.1369$, $v_{3K} = 1.043$ s$^{-1}$, $K_D = 0.958$, $K_3 = 0.522$, and $r_{5P} = 0.04$ s$^{-1}$.

The simplified model of astrocytic compartment was used only for the implementation of a cell model with unipolar morphology, and the adjacent compartments in this model were coupled as described in the Compartmental Coupling section above.

### Dynamical System Analysis

We used the simplified model to study how the dynamics of the intracellular concentrations of $Ca^{2+}$ and $IP_3$ (represented by the $c$ and $i$ variables, respectively) change with compartment radius and type of input (glutamatergic, dopaminergic, and the two together). To plot the phase-plane for an isolated compartment, we disconnected the astrocyte compartments setting the diffusion coefficients to zero. The resting condition was considered as the one without stimulation: $g = d = 0$.

To obtain an expression for the $c$-nullcline, we make $\mathrm{d}c/dt = 0$ in equation (S29). Isolating $J_{\mathrm{IP_3R}}$ in the resulting equation, we obtain:

$$J_{\mathrm{IP_3R}} = J_{\mathrm{SERCA}} - J_{\mathrm{CaERL}} - \frac{1}{\sqrt{r_{\mathrm{ER}}}} J_{\mathrm{NCX}}, \tag{S39}$$

Substituting the expression for $J_{\mathrm{IP_3R}}$ given by (S34) in this equation and writing $\frac{1}{\sqrt{r_{\mathrm{ER}}}} = \frac{\sqrt{r_{\mathrm{ER}}}}{r_{\mathrm{ER}}}$, we get:

$$\frac{FV}{A} r_C \left(\mathcal{H}_1\left(i, d_1\right)\right)^3 \left(\mathcal{H}_1\left(c, d_5\right)\right)^3 h^3 (c_{\mathrm{ER}} - c) = J_{\mathrm{SERCA}} - J_{\mathrm{CaERL}} - \frac{\sqrt{r_{\mathrm{ER}}}}{r_{\mathrm{ER}}} J_{\mathrm{NCX}}. \tag{S40}$$

Isolating $\mathcal{H}_1\left(i, d_1\right)$ in this equation gives,

$$\mathcal{H}_1\left(i, d_1\right) = \sqrt[3]{\frac{A}{VF} \frac{J_{\mathrm{SERCA}} - J_{\mathrm{CaERL}} - \frac{\sqrt{r_{\mathrm{ER}}}}{r_{\mathrm{ER}}} J_{\mathrm{NCX}}}{\left(\mathcal{H}_1\left(c, d_5\right)\right)^3 r_C h^3 \left(c_{\mathrm{ER}} - c\right)}}, \tag{S41}$$

from which we can write $i$ as:

$$i = \frac{d_1 \mathcal{H}_1\left(i, d_1\right)}{1 - \mathcal{H}_1\left(i, d_1\right)}, \tag{S42}$$

with $\mathcal{H}_1\left(i, d_1\right)$ given by (S41). This is the analytical expression of the $c$-nullcline.

The $i$-nullcline is determined by setting $\mathrm{d}i/dt = 0$ in equation (S34). However, no closed-form analytical solution exists for this nullcline. Therefore, to plot it, we employed the bisection method to numerically estimate the value of $i$ for each given value of $c$.

The Jacobian matrix of the system, used to determine the stability of the equilibrium points, is given by:

$$\mathbf{J} = \begin{bmatrix} p & q \\ r & s \end{bmatrix}, \tag{S43}$$

with

$$p = \frac{A}{VF}\alpha_{\mathrm{NCX}} + \sqrt{r_{\mathrm{ER}}}\Bigg(3\frac{r_C}{d_5}h^3\left(\mathcal{H}_1\left(c,d_5\right)\right)^2\left(\mathcal{H}_1\left(d_5,c\right)\right)^2\left(\mathcal{H}_1\left(i,d_1\right)\right)^3$$

$$\left(\beta_{\mathrm{CaER}}(r_{\mathrm{ER}}) - \left(1+\alpha_{\mathrm{CaER}}(r_{\mathrm{ER}})\right)c\right) + r_C h^3\left(\mathcal{H}_1\left(i,d_1\right)\right)^3\left(\mathcal{H}_1\left(c,d_5\right)\right)^3\left(1+\alpha_{\mathrm{CaER}}(r_{\mathrm{ER}})\right) +$$

$$r_L\left(1+\alpha_{\mathrm{CaER}}(r_{\mathrm{ER}})\right) + 4c^2 v_{\mathrm{ER}}\left(\mathcal{H}_2\left(K_{\mathrm{ER}},c\right)\right)^2\Bigg),$$

$$q = \sqrt{r_{\mathrm{ER}}}\left(3\frac{r_C}{d_1}h^3\left(\mathcal{H}_1\left(i,d_1\right)\right)^2\left(\mathcal{H}_1\left(d_1,i\right)\right)^2\left(\mathcal{H}_1\left(c,d_5\right)\right)^3\left(\beta_{\mathrm{CaER}}(r_{\mathrm{ER}}) - \left(1+\alpha_{\mathrm{CaER}}(r_{\mathrm{ER}})\right)c\right)\right),$$

$$r = v_\beta\alpha\left(K_R + K_p\frac{c}{c+K_\pi}\right)^{\alpha-1}\frac{K_p}{K_\pi g^\alpha}\left(\mathcal{H}_1\left(K_\pi,c\right)\right)^2\left(\mathcal{H}_\alpha\left(g, K_{DA} + K_p\frac{c}{c+K_\pi}\right)\right)^2 +$$

$$v_{\mathrm{DA}}\beta\left(K_R + K_p\frac{c}{c+K_\pi}\right)^{\beta-1}\frac{K_p}{K_\pi d^\beta}\left(\mathcal{H}_1\left(K_\pi,c\right)\right)^2\left(\mathcal{H}_\beta\left(d, K_{DA} + K_p\frac{c}{c+K_\pi}\right)\right)^2 +$$

$$\frac{-2v_\delta}{1+i/\kappa_\delta}\frac{c}{K_{\mathrm{PLC}\delta}^2}\left(\mathcal{H}_2\left(K_{\mathrm{PLC}\delta},c\right)\right)^2 - \frac{4v_{3K}c^3}{K_D^4}\left(\mathcal{H}_4\left(K_D,c\right)\right)^2\mathcal{H}_1\left(i,K_3\right),$$

$$s = -\frac{v_\delta/\kappa_\delta}{\left(1+i/\kappa_\delta\right)^2}\mathcal{H}_2\left(c,K_{\mathrm{PLC}\delta}\right) - \frac{v_{3K}}{K_3}\mathcal{H}_4\left(c,K_D\right)\left(\mathcal{H}_1\left(K_3,i\right)\right)^2 - r_{5\mathrm{P}},$$

# Stimulation Protocols

In all trials, compartments under glutamatergic or dopaminergic stimulation received independent presynaptic inputs modeled as Poisson spike trains with frequencies (in Hz) $\nu_g$ and $\nu_d$, respectively, for 100 s. Unless otherwise stated, glutamatergic input was applied only to the distal compartments, simulating synaptic input from a tripartite synapse [6, 7]. This corresponds to compartments 7, 8, and 9 of the unipolar morphology model (see Fig. 2**A** of the main text); compartments 7, 8, and 9 of the top process and 15, 16, and 17 of the bottom process in the bipolar morphology model (Fig. 2**B** of the main text); and compartments 7, 8, 9 of the top secondary process and 12, 13, and 14 of the bottom secondary process in the bifurcated-terminal morphology model (Fig. 2**C** of the main text). In contrast, dopaminergic input was applied to all astrocytic compartments, simulating volume transmission [8].

As a primary model response, we measured the time course of the intracellular $Ca^{2+}$ concentration in each compartment during each stimulation trial. These measurements were used to study and characterize the response of the model to stimulation by glutamate alone (with varying frequencies $\nu_g$) or dopamine alone (with varying frequencies $\nu_d$). To investigate the effects of the interaction between glutamate and dopamine, the measurements considered were the number and maximum amplitude of $Ca^{2+}$ signals for each combination of $\nu_g$ and $\nu_d$. To detect a $Ca^{2+}$ signal, we used a threshold value of $[Ca^{2+}]_{\mathrm{th}} = 0.15$ µM [10], namely whenever $[Ca^{2+}]_i \geq [Ca^{2+}]_{\mathrm{th}}$ during a simulation, a $Ca^{2+}$ signal was detected and counted. The amplitude of a $Ca^{2+}$ signal was measured as the difference between the signal peak and the baseline $Ca^{2+}$ concentration, taken as 0.073 µM. In the case of simulations using the simplified model, a $Ca^{2+}$ signal was considered whenever $c \geq c_{\mathrm{th}} = 0.4$.

In studies involving models with branched geometries, the protocol used to investigate whether glutamatergic stimulation of the distal compartments of one process could induce calcium elevations in the distal compartments of the other process consisted of applying low-frequency glutamate

stimuli ($\nu_g = 1$ Hz) to the top process and glutamate stimuli with varying frequencies to the bottom process. These studies were conducted either in the absence or presence of dopaminergic stimulation with varying frequencies $\nu_d$.

At the beginning of each stimulation trial the compartments of the astrocyte model were at their resting states. The initial values of $[\mathrm{IP_3}]$, $h$ and $[\mathrm{Ca^{2+}}]_{\mathrm{ER}}$ were calculated to ensure equilibrium in the beginning of the simulation without glutamatergic and dopaminergic stimulation ([Glu] = [DA] = 0). Similarly, the $\mathrm{Na^+}$ and $\mathrm{K^+}$ conductances ($g_{\mathrm{NaL}}$ and $g_{\mathrm{KL}}$) were calculated to impose equilibrium to $\mathrm{Na^+}$ and $\mathrm{K^+}$ concentrations [3].

In our simulations, we used the unipolar morphology model as the basic framework to investigate the emergence of compartmentalized dynamics in response to both localized (glutamatergic) and global (dopaminergic) stimulation, as well as their combined interaction. Next, we implemented a simplified compartmental version of the linear cell model to explore, through phase plane analysis, how compartment radius and neurotransmitter type influence compartmental dynamics. Finally, we returned to the detailed model to assess how astrocytic branch geometry affects these dynamics, using the models with bipolar and bifurcated-terminal morphologies. The stimulation protocols used in these experiments are outlined below.

**Local glutamatergic and global dopaminergic inputs.** We evaluated the response of the unipolar morphology model to either glutamatergic or dopaminergic stimulation. In the glutamatergic experiments, we simulated glutamatergic inputs arriving at distal compartments 7, 8 and 9. Three trials were simulated with frequencies $\nu_g = 1$ Hz, 5 Hz and 10 Hz. In the dopaminergic tests, all compartments received the dopaminergic input. We simulated four trials with frequencies $\nu_d = 0.2$ Hz, 1 Hz and 5 Hz. Responses were measured for all compartments, and the results are shown in Fig. 3 of the main text.

**Interaction of glutamatergic and dopaminergic inputs.** The unipolar morphology model received simultaneously local glutamatergic and global dopaminergic inputs. We used 13 different values for either $\nu_g$ or $\nu_d$, namely 0, 0.005, 0.01, 0.05, 0.1, 0.5, 0.75, 1, 2, 3, 4, 5 and 10 Hz, so there were 169 different combinations of $(\nu_g, \nu_d)$ values. Responses were measured in the most distal compartment of the model (compartment 9) and are summarized in the diagrams in Fig. 4 of the main text.

**Influence of compartment radius.** We studied the behavior of the simplified compartment in the phase plane for different combinations of stimuli, as a function of the compartment radius. The stimulus combinations used were: absence of stimuli, only glutamatergic stimuli, only dopaminergic stimuli, and glutamatergic and dopaminergic stimuli together. Subsequently, we constructed a version of the unipolar morphology model with the simplified compartment and studied the behaviors of the distal compartments in their respective phase planes for the same stimuli combinations mentioned above. Since each compartment has a different radius, this latter study allowed for an evaluation of the combined effect of compartment radius and stimulus type when the compartments are coupled in an astrocytic process. Results are given in Figs. 5 and 6 of the main text.

**Interaction between different processes depending on their hierarchical order.** The distal compartments (7, 8 and 9) of the bottom process were stimulated by glutamate with varying frequencies $\nu_g$ while the distal compartments (15, 16 and 17 in the bipolar morphology; 12, 13 and 14 in the bifurcated-terminal morphology) of the top process were stimulated by glutamate with a

fixed frequency of 1 Hz, which is too low to produce $Ca^{2+}$ signals. We used 17 values for $\nu_g$, namely 0, 0.005, 0.01, 0.05, 0.1, 0.5, 0.75, 1, 2, 3, 4, 5, 6, 7, 8, 9 and 10 Hz. Dopaminergic stimulation was applied to all compartments with frequency $\nu_d$ and we used the same 13 frequencies of the previous protocol. This protocol had 221 independent trials, and the results are shown in the diagrams of Fig. 7 (bipolar geometry) and Fig. 8 (bifurcated-terminal geometry) of the main text. Since the distal regions of the primary processes in the bipolar morphology model are farther apart than the distal regions of the secondary processes in the bifurcated-terminal morphology model, this experiment aimed at studying the interaction between different distal regions of an astrocyte based on their hierarchical order.

## Control Tests

To create control conditions for dopamine stimulation studies, we conducted similar experiments in which dopaminergic stimulation was replaced with glutamatergic stimulation of the soma. These experiments aimed to evaluate whether glutamatergic input at the soma could interact with distal glutamatergic stimulation to produce facilitatory effects comparable to dopamine. Specifically, we investigated (i) the interaction between somatic and distal glutamatergic stimulation in generating calcium signals in the unipolar morphology model, and (ii) the effect of somatic glutamatergic stimulation on the interaction between processes in branched morphologies. Additionally, diffusion coefficients were varied to assess their impact on interactions between glutamatergic inputs at different locations. The experimental protocols and results are presented below.

**Interaction between distal and somatic glutamatergic stimulation** Glutamate was applied to the somatic compartment at a frequency $\nu_s$ and to distal compartments 7, 8, and 9 at a frequency $\nu_g$. Both $\nu_s$ and $\nu_g$ were tested across 13 frequencies: 0, 0.005, 0.01, 0.05, 0.1, 0.5, 0.75, 1, 2, 3, 4, 5, and 10 Hz, resulting in 169 independent trials, one for each frequency combination. Unlike dopaminergic input, somatic glutamatergic stimulation did not reduce the distal glutamatergic frequency required to trigger $Ca^{2+}$ signals in compartment 9 (Fig. S6A). This suggests that the global effect of dopamine differs from the interactions between local glutamatergic inputs at different astrocytic process locations.

**Diffusion tests** To investigate how $Ca^{2+}$ and $IP_3$ diffusion affects the interaction between glutamatergic inputs at different process locations, we varied the diffusion coefficients $D_{\mathrm{Ca}}$ and $D_{\mathrm{IP}_3}$. The proximal compartment (2) was stimulated at $\nu_g = 1$ Hz, while distal compartments (7, 8, 9) were stimulated with $\nu_g$ ranging from 0.5 to 10 Hz (Fig. S7). Lower diffusion coefficients increased the number of $Ca^{2+}$ signals in distal compartment 7, while higher diffusion coefficients facilitated $Ca^{2+}$ signals in proximal and intermediate compartments (3 and 5) (Fig. S7A). However, diffusion coefficients larger than twice the default value inhibited responses in distal and intermediate compartments (5, 7, 9). Thus, reduced diffusion coefficients promoted activity in stimulated compartments, while moderate increases enhanced responses in nonstimulated compartments. Larger increases prevented $Ca^{2+}$ and $IP_3$ accumulation, reducing activity in stimulated compartments.

Since diffusion coefficients can modulate the spatial range of $Ca^{2+}$ propagation, we extended the unipolar morphology to avoid a 'ceiling effect' and further study this phenomenon. Distal compartments were stimulated with glutamate at $\nu_g = 5$ or 10 Hz, and processes with 10 and 11 compartments were considered. In the 10-compartment configuration, an additional compartment, identical to compartment 5, was inserted between compartments 5 and 6. In the 11-compartment

configuration, we added this same compartment and another, identical to compartment 7, between compartments 7 and 8. The $Ca^{2+}$ propagation distance, defined as the number of activated compartments starting from the most distal, increased with higher diffusion coefficients (Fig. S7B). Thus, the interaction between responses triggered at different sites is diffusion-dependent. Since coupling strength decreases with both diffusion coefficients and compartment length (see Eqs. S15–S17), these results suggest that longer compartments impede $Ca^{2+}$ signal propagation along the astrocytic process.

**Interaction between processes in the presence of somatic stimulation.** The soma was stimulated at frequency $\nu_s$, the distal compartments (7, 8, 9) of the bottom process with variable frequency $\nu_g$, and the distal compartments (15, 16, 17 in the bipolar morphology; 12, 13, 14 in the bifurcated-terminal morphology) of the top process at a fixed frequency of 1 Hz. Thirteen frequencies were used for $\nu_s$ (0, 0.005, 0.01, 0.05, 0.1, 0.5, 0.75, 1, 2, 3, 4, 5, 10 Hz), and 17 for $\nu_g$ (0, 0.005, 0.01, 0.05, 0.1, 0.5, 0.75, 1, 2, 3, 4, 5, 6, 7, 8, 9 and 10 Hz), resulting in 169 independent trials.

Somatic glutamatergic stimulation did not have an impact on the interaction between processes in the bipolar morphology model. Neither the number nor the amplitude of $Ca^{2+}$ signals in compartment 15 changed with the addition of glutamatergic input at the soma (Fig. 6B). In contrast, somatic glutamatergic stimulation facilitated $Ca^{2+}$ signal generation in secondary processes and enhanced their interaction in the bifurcated-terminal morphology model. The frequency required for interaction between secondary processes decreased from 2 Hz to 0.5 Hz with 1 Hz somatic stimulation (Fig. 6C). However, unlike dopaminergic stimulation, somatic input did not further lower the glutamatergic frequency required for interaction, indicating a limited facilitatory effect on interactions between secondary processes.

**Interaction between glutamate and dopamine in different branched morphologies.** Since the bipolar and bifurcated-terminal morphologies exhibited different behaviors under dopaminergic stimulation, we created models with alternative bifurcation points to investigate how bifurcation position affects the dopamine facilitatory effect. The stimulation protocol matched that of the default bifurcated-terminal morphology, with global dopaminergic input, one secondary process receiving constant glutamatergic stimulation at $\nu_g = 1$ Hz in its distal compartments, and the other secondary process receiving glutamatergic input at variable frequencies. Bifurcation points closer to the soma reduced the dopaminergic facilitation between secondary processes, resembling the behavior of the bipolar morphology when the bifurcation occurred at compartment 2 (Figs. S8A, B). In contrast, moving the bifurcation closer to the distal region enhanced the dopaminergic facilitation effect (Figs. S8C, D).

To assess whether the length of secondary processes also influences the dopamine facilitatory effect, we modified the bifurcated-terminal model by extending one secondary process (Fig. S9). This model was stimulated with global dopaminergic input, with the longer secondary process receiving constant glutamatergic stimulation at $\nu_g = 1$ Hz in its distal compartments, while the other received variable glutamatergic input. The extended secondary process reduced (Figs. S9A, B) or even abolished (Fig. S9C) the dopamine facilitatory effect, resulting in a response similar to the bipolar morphology.

# Computational and Numerical Methods

We used the amplitude of the $Ca^{2+}$ signal, defined as the $[Ca^{2+}]_i$ peak minus the resting concentration, and the number of $Ca^{2+}$ signals triggered to characterize the response of the astrocytes. In the detailed model, we counted as a $Ca^{2+}$ signal each event in which the $[Ca^{2+}]_i$ exceeds the threshold $[Ca^{2+}]_{\text{th}} = 0.15$ µM [10]. In the simplified model, we counted as a $Ca^{2+}$ signal when $c$ exceeded the threshold $c_{\text{th}} = 0.4$, defined considering the amplitude of the $Ca^{2+}$ signals simulated with the simplified model.

To calculate the numerical solution of the system of differential equations, we used the 4th-order Runge-Kutta method with time step $\mathrm{d}t = 0.01$ ms for the detailed model and the Euler method with time step $\mathrm{d}t = 0.001$ s for the simplified model. Total simulation time was $t = 100$ s. All numerical routines were implemented in Python 3.7 with the Numpy and Scipy packages. We used the Numba package with JIT compilation and GPU to speed up the simulations. To save computer memory, we sampled the time points during the simulation for the output at a rate of 0.01 Hz. Graphs were made with the Matplotlib package. Codes for the detailed model is available on GitHub at: `https://github.com/ThiagoTakechi/CompartmentalAstrocyteModel.git` and for the simplified model at: `https://github.com/ThiagoTakechi/SimplifiedAstrocyteModel.git`.

# Model Limitations

The limitations of this study must be carefully considered when interpreting the model and its outcomes. First, alternative pathways for generating $Ca^{2+}$ signals in astrocytes, as well as their modulation by dopaminergic transmission, remain unaccounted for. For instance, previous research has implicated transient receptor potential cation channels (TRPs) and mitochondria in producing localized and rapid $Ca^{2+}$ responses in astrocytic processes [11]. These mechanisms might influence the timing of astrocytic responses without significantly altering the overall effects observed in our simulations. To better account for fast, localized $Ca^{2+}$ dynamics, future extensions of the astrocyte model could incorporate these pathways, which were not included in the current version. Additionally, a previous study has shown that reactive oxygen species (ROS), generated by dopamine degradation via monoamine oxidase, can affect $Ca^{2+}$ signaling in astrocytes [12]. Dopamine also modulates astrocytic activity through interactions with the cyclic adenosine monophosphate (cAMP) pathway, which subsequently influences $Na^+$ and $K^+$ channels [13].

Activation of $D_2$ receptors has been reported to decrease intracellular $Ca^{2+}$ in hippocampal astrocytes [14]. However, studies have shown that $D_2$ receptor antagonists do not affect astrocytes in the nucleus accumbens [15], and dopamine influences prefrontal cortex astrocytes through the activation of noradrenergic $\alpha_1$ receptors [9]. Given the functional diversity of astrocytes across brain regions, it is important to note that dopamine may have varying effects on $Ca^{2+}$ signaling depending on the specific astrocyte population being studied [16]. In this study, we chose to focus on the most well-characterized mechanisms, particularly those related to glutamatergic and dopaminergic signaling, and did not incorporate $D_2$ receptor activation effects on astrocytic $Ca^{2+}$ dynamics. Expanding the model to include these additional mechanisms could provide a more comprehensive understanding of astrocytic behavior in different neural contexts.

Moreover, dopamine is not the only neuromodulator influencing intracellular $Ca^{2+}$ levels in astrocytes; noradrenaline, serotonin, and acetylcholine also contribute to modulating astrocyte activity [17, 22]. While developing our astrocyte model, we initially considered multiple pathways

through which dopamine and glutamate affect astrocytic function. However, the final model was reduced to include only the most relevant mechanisms impacting this activity.

In this model, we assumed a linear superposition of calcium fluxes from various sources, a common approach that simplifies analysis and computation. However, this assumption may limit the applicability of the model in situations where nonlinear interactions are likely, such as high-frequency stimulation or in the presence of significant spatial buffering effects [23]. Future work could address this limitation by incorporating nonlinear terms that capture more complex interactions and mechanisms.

The morphologies and parameters utilized in this study represent a simplified and generalized depiction of common features observed in astrocytes. For instance, a typical rodent protoplasmic astrocyte may exhibit 5 to 10 primary processes from which peripheral processes emanate [18]. Some studies have proposed that astrocytes possess a bushy or sponge-like structure, filling the extracellular space [19]. However, recent findings utilizing focused ion beam scanning electron microscopy (FIB-SEM) imaging have unveiled a hierarchical organization within astrocytes, consisting of fundamental elements termed cores, constrictions, and expansions [20]. The approach employed here, which models astrocytes through compartments, aligns more closely with this latter study. Furthermore, there exist physiological and morphological variances among astrocyte subtypes distributed across different brain regions [18]. However, computational limitations impose constraints on simulating astrocytes with intricate morphological architectures and associated parameters. Despite employing an idealized representation of astrocytes, the modeling and simulation techniques utilized in our study offer valuable insights into astrocyte physiology, serving as a foundation for future theoretical and experimental investigations.

# References


[1] Ullah G, Jung P, Cornell-Bell AH. Anti-phase calcium oscillations in astrocytes via inositol (1, 4, 5)-trisphosphate regeneration. Cell Calcium. 2006;39: 197-208.

[2] De Pittà M, Goldberg M, Volman V, Berry H, Ben-Jacob E. Glutamate regulation of calcium and IP3 oscillating and pulsating dynamics in astrocytes J. Biol. Phys. 2009;35: 383-411.

[3] Oschmann F, Mergenthaler K, Jungnickel E, Obermayer K. Spatial separation of two different pathways accounting for the generation of calcium signals in astrocytes PLoS Comput. Biol. 2017;13: e1005377.

[4] De Pittà M, Ben-Jacob E, Berry H. G protein-coupled receptor-mediated calcium signaling in astrocytes. In: De Pittà M, Berry H, editors. Computational glioscience. Springer Series in Computational Neuroscience. Springer, Cham; 2019. pp. 115–150

[5] Liu J, Wang F, Huang C, Long LH, Wu WN, Cai F, Wang JH, Ma LQ, Chen JG. Activation of phosphatidylinositol-linked novel D1 dopamine receptor contributes to the calcium mobilization in cultured rat prefrontal cortical astrocytes. Cell. Mol. Neurobiol. 2009;29: 317-328.

[6] Lalo U, Koh W, Lee CJ, Pankratov Y. The tripartite glutamatergic synapse. Neuropharmacology. 2021;199: 108758.

[7] Araque A, Parpura V, Sanzgiri R P, Haydon P G. Tripartite synapses: glia, the unacknowledged partner. Trends. Neurosci. 1999;22: 208–215.

[8] Liu C, Goel P, Kaeser PS. Spatial and temporal scales of dopamine transmission. Nat. Rev. Neurosci. 2021;22: 345-358.

[9] Pittolo S, Yokoyama S, Willoughby DD, Taylor CR, Reitman ME, Tse V, Wu Z, Etchenique R, Li Y, Poskanzer KE Dopamine activates astrocytes in prefrontal cortex via $\alpha$1-adrenergic receptors. Cell Rep. 2022;40: 111426.

[10] Gordleeva SY, Ermolaeva AV, Kastalskiy IA, Kazantsev VB. Astrocyte as spatiotemporal integrating detector of neuronal activity. Front. Physiol. 2019;10: 294.

[11] Ahmadpour N, Kantroo M, Stobart JL Extracellular calcium influx pathways in astrocyte calcium microdomain physiology. Biomolecules. 2021;11: 1467.

[12] Vaarmann A, Gandhi S, Abramov AY. Dopamine induces Ca2+ signaling in astrocytes through reactive oxygen species generated by monoamine oxidase. J. Biol. Chem. 2010;285: 25018-25023.

[13] Zhou Z, Ikegaya Y, Koyama R. The astrocytic cAMP pathway in health and disease. Int. J. Mol. Sci. 2019;20: 779.

[14] Jennings A, Tyurikova O, Bard L, Zheng K, Semyanov A, Henneberger C, Rusakov DA. Dopamine elevates and lowers astroglial Ca2+ through distinct pathways depending on local synaptic circuitry. Glia. 2017;65: 447–459.

[15] Corkrum M, Covelo A, Lines J, Bellocchio L, Pisansky M, Loke K, Quintana R, Rothwell PE, Lujan R, Marsicano G, Martin ED. Dopamine-evoked synaptic regulation in the nucleus accumbens requires astrocyte activity Neuron. 2020;105: 1036-1047.

[16] Chai H, Diaz-Castro B, Shigetomi E, Monte E, Octeau JC, Yu X, Cohn W, Rajendran PS, Vondriska T, Whitelegge JP, Coppola G, Khakh BS. Neural circuit-specialized astrocytes: transcriptomic, proteomic, morphological, and functional evidence Neuron. 2017;95: 531–549.

[17] Pacholko AG, Wotton CA, Bekar LK. Astrocytes—the ultimate effectors of long-range neuromodulatory networks?. Front. Cell. Neurosci. 2020;14: 581075.

[18] Verkhratsky A, Nedergaard M. Physiology of astroglia Physiol. Rev. 2018;98: 239-389.

[19] Aten S, Kiyoshi CM, Arzola EP, Patterson JA, Taylor AT, Du Y, Guiher AM, Philip M, Camacho EG, Mediratta D, Collins K. Ultrastructural view of astrocyte arborization, astrocyte-astrocyte and astrocyte-synapse contacts, intracellular vesicle-like structures, and mitochondrial network. Prog Neurobiol. 2022;213: 102264.

[20] Salmon CK, Syed TA, Kacerovsky JB, Alivodej N, Schober AL, Sloan TF, Pratte MT, Rosen MP, Green M, Chirgwin-Dasgupta A, Mehta S. Organizing principles of astrocytic nanoarchitecture in the mouse cerebral cortex. Curr Biol. 2023;33: 957-972.

[21] Goldberg M, De Pittà M, Volman B, Berry H, Ben-Jacob, E. Nonlinear gap junctions enable long-distance propagation of pulsating calcium waves in astrocyte networks. PLoS Comp. Biol. 2010; 6:e1000909.

[22] Rangel-Gomez M, Alberini CM, Deneen B, Drummond GT, Manninen T, Sur M, Vicentic A. Neuron–glial interactions: implications for plasticity, behavior, and cognition J. Neurosci. 2024; 44: e1231242024

[23] Sala F, Hernández-Cruz, A. Calcium diffusion modeling in a spherical neuron. Relevance of buffering properties. Biophys. J. 1990; 57:313-324.
