# Supplementary material for: Dopamine facilitates the response to glutamatergic inputs in astrocyte cell models
Source: PLoS Comput Biol. 2024 Dec 16;20(12):e1012688. doi: 10.1371/journal.pcbi.1012688 (PMC11684655; doi:10.1371/journal.pcbi.1012688)
Supplement: S1 Table — Tables listing all parameters of the detailed and simplified models. (PDF) [file pcbi.1012688.s002.pdf]

Dopamine facilitates the response to glutamatergic inputs in astrocyte cell models
S1 Tables: Model parameters

Thiago Ohno Bezerra, Antonio C. Roque

Table A: Detailed Model Parameters.

Parameter	Value	Unit	Reference
Resting State			
$[Ca^{2+}]_i$	0.073	µM	[10]
$[Ca^{2+}]_{ER}$	23.3480	µM	—
$[Ca^{2+}]_e$	1800	µM	[10]
$[Na^+]_i$	15000	µM	[10]
$[Na^+]_e$	145000	µM	[10]
$[K^+]_i$	100000	µM	[10]
$[K^+]_e$	3000	µM	[10]
$[IP_3]$	0.1917	µM	—
h	0.8028	—	—
v	-85	mV	[10]
[Glu]	0	µM	—
[DA]	0	µM	—
IP_3 Dynamics			
PLCβ Synthesis			
K_p	10	µM	[1]
K_π	0.6	µM	[1]
PLCδ Synthesis			
v_δ	0.025	µM/s	[3]
κ_δ	1.5	µM	[1]
$K_{PLC\delta}$	0.1	µM	[1]
IP_3-3K Degradation			
v_{3K}	2	µM/s	[1]
K_D	0.7	µM	[1]
K_3	1	µM	[1]
IP-5P Degradation			
r_{5P}	0.04	1/s	[1]
Glutamate Transmission			

ρ_{Glu}	0.5	µM	—
G_{Glu}	100	1/s	[8]
K_R	1.3	µM	[1]
v_β	0.674	µM/s	—
α	0.7	—	[1]
Dopamine Transmission			
ρ_{DA}	3	µM	[2]
G_{DA}	4.201	1/s	[4]
v_{DA}	0.025	µM/s	[6]
K_{DA}	5	µM	[6]
β	0.5	—	[6]
ER Leak Current			
r_L	0.11	1/s	[1]
SERCA Current			
v_{ER}	11.93	µM/s	[3]
K_{ER}	0.1	µM	[3]
h Dynamics			
d_1	0.13	µM	[1]
d_5	0.08234	µM	[1]
d_2	1.049	µM	[1]
d_3	0.9434	µM	[1]
a_2	0.2	1/(µM s)	[1]
r_C	6	1/s	[1]
GluT			
J_{GluTmax}	0.68	pA/µm^2	[10]
K_{GluTmN}	15000	µM	[10]
K_{GluTmK}	5000	µM	[10]
K_{GluTmg}	34	µM	[10]
NKA			
J_{NKAmax}	1.52	pA/µm^2	[10]
K_{NKAmN}	10000	µM	[10]
K_{NKAmK}	1500	µM	[10]
NCX			
J_{NCXmax}	0.0001	pA/µm^2	[10]
K_{NCXmN}	87.5	µM	[10]
K_{NCXmC}	1.380	µM	[10]
k_{sat}	0.1	—	[10]
η	0.35	—	[10]
Voltage Parameter			
C_m	0.01	F/m^2	[10]
Leak Currents			
g_{NaL}	13.482808	S/m^2	[10]

E_{Na}	61	mV	[10]
g_{KL}	145.814171	S/m^2	[10]
E_{K}	-94	mV	[10]
Diffusion Coefficients			
D_{Ca}	0.2	µm^2/s	[11]
D_{CaER}	0.001	µm^2/s	[3]
$D_{\mathrm{IP_3}}$	0.2	µm^2/s	[11]
D_{Na}	0.316	µm^2/s	[7]
D_{K}	0.938	µm^2/s	[7]
D_{Cao}	4.52	µm^2/s	[5]
D_{Nao}	26.6	µm^2/s	[7]
D_{Ko}	1.732	µm^2/s	[7]
D_{glu}	4e-4	µm^2/s	[11]
D_{DA}	13.8	µm^2/s	[9]
Physical Constants			
T	303.16	K	—
F	96500	C/mol	—
R	8.314	J/mol.K	—
Ca^{2+} Threshold			
$[Ca^{2+}]_{\mathrm{th}}$	0.15	µM	[3]

The resting state values of $[Ca^{2+}]_{\mathrm{ER}}$, $[IP_3]$ and h were calculated to impose stable equilibrium. We set the resting extracellular concentrations of [Glu] and [DA] to zero. The value of G_{Glu} was adjusted considering the decay time observed in [8]. The values of ρ_{Glu} and v_β were adjusted to produce a similar response to that observed in [1, 3] using the glutamate concentration described there. The value of ρ_{DA} was adjusted to reproduce the extracellular dopamine concentration in the medial prefrontal cortex after stimulation (60 Hz, 2 s) of the VTA [2]. The value of G_{DA} was adjusted to reproduce the dopaminergic decay time in the prefrontal cortex [4]. Using the parameters mentioned above, v_{DA}, K_{DA} and β were adjusted to reproduce the duration and amplitude of Ca^{2+} signals triggered by dopamine [6]. The values of v_{ER} and K_{ER} were adjusted according to the code kindly provided to us by the authors of [3] to reproduce the results of their paper. The value of J_{NCXmax} was adjusted to ensure that glutamatergic stimulation of the distal compartments triggers Ca^{2+} signals in the neighboring compartments (Fig S5). Lower values resulted in currents that did not alter the astrocyte response. The values of g_{NaL} and g_{KL} were adjusted to impose equilibrium to $[Na^+]_i$, and $[K^+]_i$ in the resting state (equations (5)–(8)) of the main text.

The diffusion coefficients were obtained from [7] considering the values given for the brain and nerve tissues. They were divided by a factor of 50 to match the values given in the code of [3] (kindly provided to us by the authors). With this change, we were able to reproduce the simulation results of [3].

Table B: Simplified Model Parameters.

Parameter	Value	Unit
Resting State		
c	0.1	—
i	0.1	—
d	0	—
g	0	—
h	0.8	—
IP_3 Dynamics		
PLCβ Synthesis		
K_p	0.080	—
K_π	0.821	—
PLCδ Synthesis		
v_δ	0.013	1/s
κ_δ	0.782	—
$K_{\mathrm{PLC}\delta}$	0.1369	—
IP_3-3K Degradation		
v_{3K}	1.043	1/s
K_D	0.958	—
K_3	0.522	—
IP-5P Degradation		
r_{5P}	0.04	1/s
Glutamate Transmission		
ρ_g	$0.4 \cdot 10^{-3}$	—
G_g	100	1/s
K_R	$0.104 \cdot 10^{-2}$	—
v_β	0.211	1/s
α	0.7	—
Dopamine Transmission		
ρ_d	$1 \cdot 10^{-3}$	—
G_d	4.201	1/s
v_{DA}	0.013	1/s
K_{DA}	$5 \cdot 10^{-3}$	—
β	0.5	—
ER Leak Current		
r_L	0.11	1/s
SERCA Current		
v_{ER}	18.782	1/s
K_{ER}	0.1164	—
IP_3R Current		
d_1	0.0646	—
d_5	0.1074	—

r_C	6.0	1/s
NCX		
α_{NCX}	$1.169 \cdot 10^{-4}$	A/mM.m^2
β_{NCX}	$1.234 \cdot 10^{-5}$	A/mM.m^2
Diffusion Constants		
D_c	0.3	1/s
D_i	0.3	1/s
D_g	$4 \cdot 10^{-4}$	1/s
D_d	13.8	1/s
Ca^{2+} Threshold		
c_{th}	0.4	—

In the simplified model, the variables $[\text{Ca}^{2+}]_i$ and $[\text{IP}_3]$ were rescaled to dimensionless variables c and i, respectively. This was done by multiplying them by the factors $\lambda = 1.37$ mM^{-1} and $\eta = 0.522$ mM^{-1}, respectively, so that their resting values are both equal to 0.1 (more details are given in the S1 Text). The rescaling of the extracellular glutamate and dopamine concentrations was done by their average values at 5 Hz inputs. The remaining parameters were adjusted to reproduce the detailed model response to glutamatergic and dopaminergic stimulations.

References

[1] De Pittà M, Goldberg M, Volman V, Berry H, Ben-Jacob E. Glutamate regulation of calcium and IP3 oscillating and pulsating dynamics in astrocytes J. Biol. Phys. 2009;35: 383-411.

[2] Garris PA, Collins LB, Jones SR, Wightman RM Evoked extracellular dopamine in vivo in the medial prefrontal cortex J. Neurochem. 1993; 61: 637–647

[3] Gordleeva SY, Ermolaeva AV, Kastalskiy IA, Kazantsev VB. Astrocyte as spatiotemporal integrating detector of neuronal activity. Front. Physiol. 2019;10: 294.

[4] Holloway ZR, Freels TG, Comstock JF, Nolen HG, Sable HJ, Lester DB Comparing phasic dopamine dynamics in the striatum, nucleus accumbens, amygdala, and medial prefrontal cortex Synapse. 2019; 73: e22074

[5] Calcium diffusion enhanced after cleavage of negatively charged components of brain extracellular matrix by chondroitinase ABC Calcium diffusion enhanced after cleavage of negatively charged components of brain extracellular matrix by chondroitinase ABC. J Physiol. 2009; 587: 4029–4049.

[6] Liu C, Goel P, Kaeser PS. Spatial and temporal scales of dopamine transmission. Nat. Rev. Neurosci. 2021;22: 345-358.

[7] McLennan H. The diffusion of potassium, sodium, sucrose and inulin in the extracellular spaces of mammalian tissues. BBA. 1957;24:1–8.

[8] Nevalainen N, Lundblad M, Gerhardt GA, Strömberg I. Striatal glutamate release in L-DOPA-induced dyskinetic animals PLoS One. 2013;8: e55706.

[9] Nicholson C. Interaction between diffusion and Michaelis-Menten uptake of dopamine after iontophoresis in striatum. Biophys. J. 1995; 68: 1699–1715.

[10] Oschmann F, Mergenthaler K, Jungnickel E, Obermayer K. Spatial separation of two different pathways accounting for the generation of calcium signals in astrocytes PLoS Comput. Biol. 2017;13: e1005377.

[11] Verisokin AY, Verveyko DV, Postnov DE, Brazhe AR. Modeling of astrocyte networks: toward realistic topology and dynamics. Front. Cell. Neurosci. 2021;15: 645068.
